# Phosphorylation of AGO2 by TBK1 Promotes the Formation of Oncogenic miRISC in NSCLC

**DOI:** 10.1002/advs.202305541

**Published:** 2024-02-13

**Authors:** Xian Zhao, Yingting Cao, Runhui Lu, Zihan Zhou, Caihu Huang, Lian Li, Jiayi Huang, Ran Chen, Yanli Wang, Jian Huang, Jinke Cheng, Junke Zheng, Yujie Fu, Jianxiu Yu

**Affiliations:** ^1^ Department of Biochemistry and Molecular Cell Biology, Shanghai Key Laboratory of Tumor Microenvironment and Inflammation Shanghai Jiao Tong University School of Medicine Shanghai 200025 China; ^2^ Department of Thoracic Surgery, Ren Ji Hospital Shanghai Jiao Tong University School of Medicine Shanghai 200120 China; ^3^ Department of Pathophysiology, Key Laboratory of Cell Differentiation and Apoptosis of Chinese Ministry of Education Shanghai Jiao Tong University School of Medicine Shanghai 200025 China

**Keywords:** AGO2, Gefitinib, miRISC, non‐small‐cell lung cancer (NSCLC), TBK1

## Abstract

Non‐small‐cell lung cancer (NSCLC) is a highly lethal tumor that often develops resistance to targeted therapy. It is shown that Tank‐binding kinase 1 (TBK1) phosphorylates AGO2 at S417 (pS417‐AGO2), which promotes NSCLC progression by increasing the formation of microRNA‐induced silencing complex (miRISC). High levels of pS417‐AGO2 in clinical NSCLC specimens are positively associated with poor prognosis. Interestingly, the treatment with EGFR inhibitor Gefitinib can significantly induce pS417‐AGO2, thereby increasing the formation and activity of oncogenic miRISC, which may contribute to NSCLC resistance to Gefitinib. Based on these, two therapeutic strategies is developed. One is jointly to antagonize multiple oncogenic miRNAs highly expressed in NSCLC and use TBK1 inhibitor Amlexanox reducing the formation of oncogenic miRISC. Another approach is to combine Gefitinib with Amlexanox to inhibit the progression of Gefitinib‐resistant NSCLC. This findings reveal a novel mechanism of oncogenic miRISC regulation by TBK1‐mediated pS417‐AGO2 and suggest potential therapeutic approaches for NSCLC.

## Graphical abstract

Jianxiu Yu and colleagues show that TBK1 phosphorylates AGO2, which promotes NSCLC development by increasing the activity of oncogenic miRNAs. They use a TBK1 inhibitor Amlexanox to interfere the formation of multiple oncogenic miRISCs and overcome NSCLC resistance to EGFR‐TKI. Their study reveals a new mechanism of miRNA regulation and a potential treatment for drug‐resistant NSCLC.

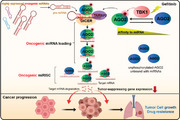

## 1 Introduction

Lung cancer, especially non‐small cell lung cancer (NSCLC), remains the leading cause of death worldwide.^[^ ^1^ ^]^ Although advanced therapies have been applied for the treatment of lung cancer, there is still no effective therapeutic strategy for some NSCLC patients. Moreover, the prolongation of overall survival is also limited due to drug resistance.^[^ ^2^ ^]^ Patients with epidermal growth factor receptor (EGFR) mutations have seen little improvement in overall survival from tyrosine kinase inhibitors.^[^ ^3^, ^4^, ^5^, ^6^ ^]^ The major reason for the failure of currently available therapeutic approaches is the development of drug resistance. This resistance is associated with gene mutations, cancer stem cells, overexpression of oncogenes, deletion or inactivation of tumor suppressor genes,^[^ ^7^, ^8^, ^9^, ^10^ ^]^ and other mechanisms that have not yet been determined.

RNA‐based therapeutics is a promising field that has gained a lot of attention recently. They can target specific genes and silence them, which can enhance the effects of chemotherapy and immunotherapy.^[^ ^11^, ^12^, ^13^, ^14^ ^]^ Compared to conventional drugs, RNA‐based therapies including antisense oligonucleotides (ASOs), small interfering RNAs (siRNAs), and microRNAs (miRNAs), have many advantages, such as high specificity, low toxicity, and easy delivery.^[^ ^15^, ^16^ ^]^ MiRNAs are endogenous small non‐coding RNAs involved in gene expression regulation at the posttranscriptional level through degradation of target messenger RNAs (mRNAs) or inhibition of translation. They play diverse roles in various physiological and pathophysiological progressions, including tumorigenesis and progression,^[^ ^17^, ^18^ ^]^ cell‐cycle regulation,^[^ ^19^ ^]^ cell metabolism,^[^ ^20^ ^]^ and immune response.^[^ ^21^ ^]^ It is highly noteworthy that oncogenic miRNAs such as miR‐21 are highly expressed in NSCLC and participate in the occurrence, development, and metastasis of tumors.^[^ ^22^, ^23^, ^24^, ^25^ ^]^ Therefore, anti‐oncogenic miRNA therapy is a potential and effective treatment method. Moreover, recent studies have indicated that some oncogenic miRNAs contribute to the resistance of EGFR mutated NSCLC tumors to TK inhibitors.^[^ ^26^, ^27^ ^]^ Therefore, a miRNA‐based combination therapeutic strategy is promising and beneficial for improving therapeutic response. The key challenge is to explore effective target miRNAs and combination approaches.

In human, primary miRNA is initially processed into precursor miRNA by DROSHA/DGCR8, and the latter is further processed into a miRNA duplex by DICER/TARBP2.^[^ ^28^ ^]^ Once the miRNA duplex is recruited to AGO2, these two strands are unwound, thus starting a mature miRNA‐induced silencing complex (miRISC) formation.^[^ ^29^ ^]^ The guide strand of miRNA loaded into AGO2 leads to the formation of effective miRISCs, which regulate gene expression by targeting mRNAs, while the passenger strand of miRNA is degraded or recycled.^[^ ^30^, ^31^, ^32^, ^33^, ^34^, ^35^ ^]^ The miRISC formation is influenced by various factors, such as the thermodynamic stability, sequence and structure of the miRNA duplex.^[^ ^36^ ^]^ The miRISC loading complex (miRLC) consists of AGO2, DICER and TARBP2 proteins and facilitates miRISC formation.^[^ ^30^, ^31^, ^32^ ^]^ Our previous studies have shown that SUMOylation of TARBP2 promotes AGO2 and pre‐miRNA loading into miRLC, resulting in the formation of effective miRISC.^[^ ^32^ ^]^ We also found that hypoxia‐induced Met1‐linked linear ubiquitination of AGO2 suppresses miRNA‐targeted mRNA recruiting to AGO2, and thereby decreasing miRNA efficiency.^[^ ^37^ ^]^ However, the detailed mechanism of how a mature miRNA is loaded into AGO2 and whether the unknown key modification of AGO2 is involved in miRISC formation and miRNA activity are still unclear.

Tank‐binding kinase 1 (TBK1) and its homologue IKKε are serine/threonine kinases that activate type I IFN and antiviral immunity by phosphorylating IRF3 and IRF7.^[^ ^38^ ^]^ Upon viral infection, the stimulator of interferon genes (STING) binds with TBK1, thus to enhance the phosphorylation of TBK1 at S172 (pS172‐TBK1), which is located within the classical kinase activation loop of TBK1.^[^ ^39^ ^]^ TBK1 is also involved in cancer progression,^[^ ^40^ ^]^ but its mechanism is unclear. We showed that TBK1 phosphorylates AGO2 and regulates the formation and activity of miRISC. The high phosphorylation level of AGO2 is associated with poor prognosis in NSCLC patients and drug resistance. Targeting AGO2 phosphorylation may offer a potential novel therapeutic strategy for NSCLS.

## 2 Results

### 2.1 TBK1 Promotes miRNA‐Guided Gene Silencing by Directly Interacting with AGO2

**Figure 1:**
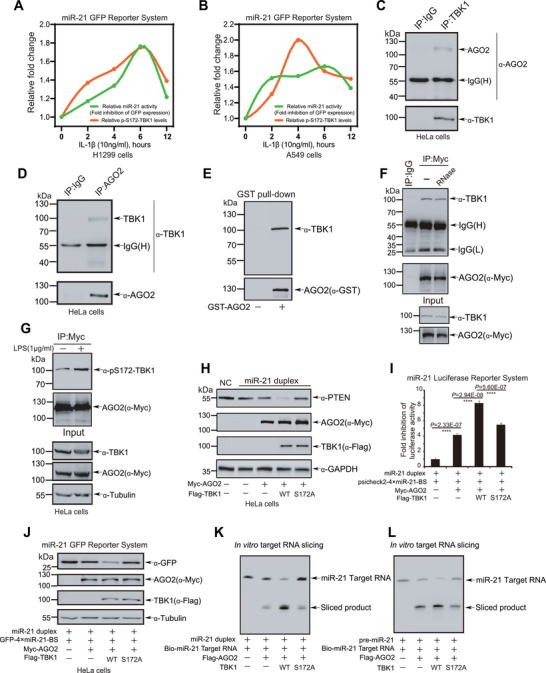
TBK1 directly interacts with AGO2 and promotes miRNA‐guided gene silencing. A,B) H1299 A) or A549 B) cells transfected with GFP‐4×miR‐21‐BS were treated with IL‐1β (10 ng ml^−1^) for the indicated time, and then harvested for the miR‐21 miRISC GFP reporter assay. C) HeLa cell lysates were used for Co‐IP with anti‐TBK1 antibody, and followed by Western blotting analysis with anti‐AGO2 antibody. D) HeLa cell lysates were used for Co‐IP with anti‐AGO2 antibody, and followed by Western blotting analysis with anti‐TBK1 antibody. E) Purified GST‐AGO2 was incubated with HeLa cell lysates for pull‐down assay. F) Lysates form 293T cells transfected with Myc‐AGO2 were treated with RNase (100 ng ml^−1^) for 30 min at 37 °C, and then used for Co‐IP with anti‐Myc antibody, and followed by Western blotting analysis with anti‐TBK1 antibody. G) HeLa cells transfected with Myc‐AGO2 were treated with LPS (1 µg ml^−1^) for 12 h before harvested. Lysates were used for IP with anti‐Myc antibody, and followed by Western blotting analysis with pS172‐TBK1 antibody. H) Lysates form HeLa cells co‐transfected miR‐21 duplex, Myc‐AGO2 with Flag‐TBK1^WT^ or TBK1^S172A^ were used for Western blotting analysis with anti‐PTEN antibody. I) 293T cells co‐transfected miR‐21 duplex mimics, psiCHECK2‐4×miR21‐BS, Myc‐AGO2 with Flag‐TBK1^WT^ or TBK1^S172A^ were harvested for the dual‐luciferase activity assay. The Renilla luciferase values were normalized to the Firefly luciferase activity and plotted as relative luciferase activity. Data were presented as mean ± SD, n = 3. Statistical analysis was performed using one‐way ANOVA. ****p < 0.0001. J) HeLa cells co‐transfected miR‐21 duplex mimics, GFP‐4×miR‐21‐BS, Myc‐AGO2 with Flag‐TBK1^WT^ or TBK1^S172A^ were harvested for Western blotting analysis with anti‐GFP antibody. K‐L) Lysates from 293T cells co‐transfected Flag‐AGO2 with TBK1^WT^ or TBK1^S172A^ were immunoprecipitated by Flag‐AGO2, and then this purified AGO2 protein were co‐incubated with miR‐21 duplex K) or pre‐miR‐21 L) and biotin‐tagged miR‐21 target for in vitro target RNA slicing assay. The cleavage products were detected on 20% urea PAGE by Northern blotting analysis.

To easily detect miRNA activity, we generated the miR‐21‐GFP Reporter System (the plasmid named as GFP‐4×miR21‐BS), in which four repetitive miR21 binding site sequences were constructed into the 3′‐untranslated region (3′‐UTR) of *GFP* gene (Figure S1A, Supporting Information). To investigate whether TBK1 is involved in the regulation of miRNA activity, H1299 and A549 cells transfected GFP‐4×miR21‐BS plasmid were treated with IL‐1β for indicated times, and then harvested for Western blotting (WB) analysis on the levels of GFP protein and pS172‐TBK1. We found that both pS172‐TBK1 and miR‐21 activity were in parallel increased over time in response to IL‐1β stimulation (**Figure** **1**A,B; Figure S1B–D, Supporting Information), indicating that TBK1 is potentially involved in the regulation of miRNA activity. Therefore, first of all, we wanted to know whether AGO2 has physical interaction with TBK1. Lysates from HeLa cells were used for reciprocal co‐immunoprecipitation (Co‐IP) with either anti‐TBK1 (Figure 1C) or anti‐AGO2 antibody (Figure 1D), and followed by WB analysis to show that endogenous AGO2 indeed interacted with TBK1. Further to confirm the direct interaction between AGO2 and TBK1, bacterially expressed glutathione S‐transferase (GST)‐tagged AGO2 protein was incubated with lysates from HeLa cells for GST pull‐down assay, which showed that TBK1 directly bound to AGO2 (Figure 1E). Considering that AGO2 is an RNA binding protein, we further tested whether the interaction between AGO2 and TBK1 depends on RNA. Indeed, the result confirmed that this interaction was independent of RNA (Figure 1F). TBK1 is activated with pS172‐TBK1 by lipopolysaccharide (LPS) and pathogen nucleic acids which trigger the activation of TLR3/4 and cGAS‐STING signaling axis.^[^ ^39^ ^]^ Interestingly, the interaction of AGO2 and the active form pS172‐TBK1 in HeLa cells was significantly enhanced after LPS treatment (Figure 1G).

Next, we attempted to explore whether TBK1 influences the function of AGO2 mediating miRNA‐guided gene silencing. We generated an inactive mutant of TBK1 with a substitution of alanine for serine at position 172 (S172A),^[^ ^41^ ^]^ which abrogates its auto‐phosphorylation and activation. We then co‐transfected the miR‐21 or let‐7a mimics, Myc‐AGO2, and Flag‐TBK1^WT^ or Flag‐TBK1^S172A^ into HeLa cells, and then determined the expression level of PTEN or HMGA2 by WB analysis, because *PTEN* and *HMGA2* are direct target of miR‐21^[^ ^42^ ^]^ and let‐7,^[^ ^43^ ^]^ respectively. The results showed that AGO2 enhanced the inhibitory effect of miR‐21 or let‐7a on the expression of PTEN or HMGA2, respectively, which was greatly augmented by TBK1^WT^, but not by TBK1^S172A^ (Figure 1H; Figure S1D, Supporting Information). Moreover, we employed miR‐21 or let‐7 miRISC luciferase reporter assay, in which four repetitive miR21 or let‐7a binding site (BS) sequences was reconstructed into the 3′‐UTR of *Renilla* luciferase in the psiCHECK2 vector getting a reporter construct psiCHECK2‐4×miR21/let‐7a‐BS, and the miRISC luciferase activity was normalized by *Firefly* luciferase. Above construct was co‐transfected together with the miR‐21 or let‐7a mimics, AGO2 and TBK1^WT^ or TBK1^S172A^ into 293T cells, and the reporter activity was measured. The luciferase assays showed that AGO2 significantly increased the inhibition of the luciferase reporter activities, which could be significantly enhanced by TBK1^WT^, but not by TBK1^S172A^ (Figure 1I; Figure S1H, Supporting Information). To further confirm this, we co‐transfected GFP‐4×miR21/let‐7a‐BS, AGO2 and TBK1^WT^ or TBK1^S172A^ into HeLa cells, and the GFP protein level was detected by WB analysis. As expectedly, the similar results were obtained that the GFP expression level was suppressed AGO2, and this effect was more pronounced by TBK1^WT^, but not by TBK1^S172A^ (Figure 1J; Figure S1F, Supporting Information)_._

In miRISC silencing, AGO2 are a crucial component that is directed to the target mRNAs through base pairing with miRNA. Full complementarity between siRNA/miRNA and its target mRNA results in AGO2‐mediated cleavage, commonly referred to as “slicing” of the target mRNA.^[^ ^44^ ^]^ To examine whether TBK1 plays a role in AGO2‐mediated gene slicing, we designed a 5′‐biotin‐miR‐21 target RNA containing native miR‐21‐targeted sequences (Figure S1G, Supporting Information). Lysates from 293T cells co‐transfected Flag‐AGO2 with TBK1^WT^ or TBK1^S172A^ were Co‐IPed with anti‐Flag antibody, then AGO2 complexes were purified with 3xFlag peptide. The biotin‐miR‐21 target RNA, miR‐21 duplex (Figure 1K) or pre‐miR‐21 (Figure 1L) were incubated with/without AGO2 complexes for the in vitro target RNA slicing assay. The results showed that in the reactions without AGO2 complexes, no cleavage product was observed. As expectedly, TBK1^WT^ increased target RNA cleavage with miR‐21 or pre‐miR‐21, while TBK1^S172A^ mutant had no effect in the level of cleavage products, indicating that TBK1 promotes AGO2‐mediated miRNA‐guided gene slicing in vitro. Taken together, all above results reveal that TBK1 directly interacts with AGO2 and promotes miRNA‐guided gene silencing dependent on its kinase activity.

### 2.2 TBK1 Promotes miRNA Loading to AGO2 to Form Effective miRISC

**Figure 2:**
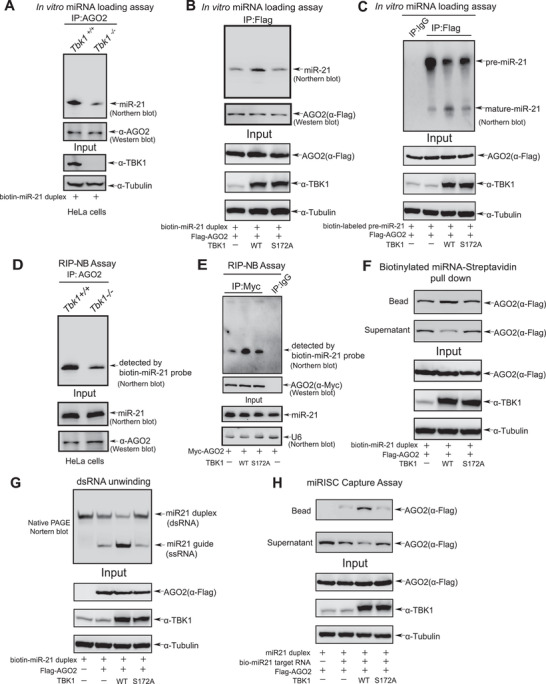
TBK1 promotes miRNAs loading and miRISC formation. A) Lysates from HeLa‐*Tbk1^+/+^* and HeLa‐*Tbk1^−/‐^* cells were immunoprecipitated by AGO2, and then the beads coupled with AGO2 were co‐incubated with biotin‐miR‐21 duplex for in vitro miRNA loading assay. The miR‐21 associated with AGO2 was detected by Northern blotting analysis. B‐C) Lysates from 293T cells co‐transfected Flag‐AGO2 with TBK1^WT^ or TBK1^S172A^ were immunoprecipitated by Flag‐AGO2, then the beads were co‐incubated with purified biotin‐miR‐21 duplex B) or pre‐miR‐21 C) for in vitro miRNA loading assay. The miR‐21 associated with AGO2 was detected by Northern blotting analysis. D) HeLa‐*Tbk1^+/+^* and HeLa‐*Tbk1^−/−^* cells were lysed by for the RIP‐NB assay with anti‐AGO2 antibody, and then endogenous miR‐21 associated with AGO2 was detected by Northern blotting analysis with biotin‐miR‐21 probe. E) 293T cells co‐transfected Myc‐AGO2 with Flag‐TBK1^WT^ or TBK1^S172A^ were lysed by for the RIP‐NB assay with anti‐Myc antibody, and then endogenous miR‐21 associated with AGO2 was detected by Northern blotting analysis with biotin‐miR‐21 probe. F) Lysates from 293T cells co‐transfected Flag‐AGO2 with TBK1^WT^ or TBK1^S172A^ were incubated with streptavidin‐Dynabeads‐coupled‐biotinylated miR‐21 duplex for biotinylated RNA‐streptavidin pull down assay. AGO2 pulled down by miR‐21 on the beads and not pulled down by miR‐21 in the supernatant were examined by Western blotting analysis with anti‐Flag antibody. G) Flag‐AGO2 purified from 293T cells co‐transfected Flag‐AGO2 with TBK1^WT^ or TBK1^S172A^ using 3×Flag peptide was incubated with miR‐21 duplex mimics for miR‐21 unwinding assays. The single‐stranded (ss) RNA molecules unwinding from double‐stranded (ds) miR‐21 substrates was detected on native polyacrylamide gels. H) Lysates from 293T cells co‐transfected Flag‐AGO2 with TBK1^WT^ or TBK1^S172A^ were co‐incubated with miR‐21 duplex and biotin‐tagged miR‐21 target RNA. Complex of AGO2, miR‐21, and miR‐21 target RNA was pulled down by streptavidin beads, and then detected by Western blotting analysis with anti‐Flag antibody.

Next, we investigated the mechanism by which TBK1 modulates the efficiency of miRNA‐mediated gene silencing by miRISC. The formation of mature miRISC involves at least two steps: the loading of the miRNA duplex (miRNA/miRNA*) onto AGO2, followed by the unwinding of miRNA duplex and AGO2 selecting the guide strand within miRISC.^[^ ^29^, ^45^ ^]^ Therefore, we first performed the in vitro miRNA loading assays to determine whether TBK1 participates in the process of miRNA loading onto AGO2. Lysates from TBK1‐knockout (by an CRISPR‐Cas9 system) HeLa cells were immunoprecipitated (IP‐ed) by anti‐AGO2 antibody, and then the protein A/G beads were co‐incubated with purified biotin‐miR‐21 duplex. The guide strand loaded onto AGO2 detected by Northern blotting (NB) was significantly reduced in TBK1 deficiency (**Figure** **2**A). Similarly, by using biotin‐labelled pre‐let‐7a‐3, the binding of mature let‐7a to AGO2 was significantly reduced by TBK1 knockdown (with shRNA) (Figure S2A, Supporting Information) whereas greatly enhanced by overexpression of TBK1 (Figure S2B, Supporting Information). These results suggest that TBK1 can promote miRNA loading to AGO2. Further to investigate whether this effect of TBK1 is dependent on its kinase activity, we performed the similar in vitro miRNA loading assays by using lysates from 293T cells co‐transfected TBK1^WT^ or inactive mutant TBK1^S172A^ and Flag‐AGO2 for IP. Purified biotin‐miR‐21 duplex (Figure 2B) and biotin‐labelled pre‐miR‐21 (Figure 2C) were added in the IP, respectively. The guide strand /mature miR‐21 loaded onto AGO2 was detected by NB, showing that the binding of mature miR‐21 to AGO2 was significantly increased by TBK1^WT^ but not by TBK1^S172A^ in vitro (Figure 2B,C). In addition, we also performed in vivo RIP‐NB experiments and obtained the same results, that is, TBK1 deficiency decreased (Figure 2D) whereas TBK1 overexpression increased endogenous miR‐21 (Figure 2E) or miR‐let‐7a (Figure S2C, Supporting Information) loading into AGO2 of miRISC, in which its kinase activity is required (Figure 2E).

To validate TBK1 promoting miRISC formation, cell lysates from 293T cells transiently expressing Flag‐AGO2 and TBK1^WT^ or TBK1^S172A^ were incubated with streptavidin‐Dynabeads‐coupled‐biotinylated miR‐21 duplex mimics for the pull‐down assays, showing that AGO2 (in beads) recruited to miR‐21 mimics was significantly increased by TBK1^WT^ but not by TBK1^S172A^ (Figure 2F). We also performed a unwinding assay of miR‐21 duplex mimics in vitro, and showed that single‐stranded RNA (ssRNA) molecules unwound from the double‐stranded RNA (dsRNA) of miR‐21 duplex mimics were significantly increased by TBK1^WT^ but not by TBK1^S172A^ (Figure 2G), indicating that TBK1 facilitated AGO2‐mediated the unwinding of miRNA duplex. Moreover, we performed an RISC‐capture assay, in which the biotin‐tagged miR‐21 target RNA was incubated with cell lysates from 293T cells co‐transfected with indicated plasmids and miR‐21 duplex mimics to pull down the activated mature miRISC. The results showed that the AGO2:miRNA complexes captured by biotin‐tagged miR‐21 targeted RNA were strongly enhanced by TBK1^WT^ but not by TBK1^S172A^, which suggested TBK1 promoting miRISC formation (Figure 2H). To further confirm that the kinase activity of TBK1 is particularly required for miRNA loading and miRISC formation, we introduced the constitutive active form of myr‐AKT1 featuring N‐terminal attached myristoylation signal into the experiments. Flag‐AGO2 was co‐transfected with TBK1^WT^, TBK1^S172A^, or TBK1^S172A^/myr‐AKT1 into 293T cells. Lysates were used for in vitro miRNA loading assay (Figure S2D, Supporting Information) and a biotinylated miRNA‐Streptavidin pull down assay (Figure S2E, Supporting Information). The results showed that the loading of mature miR‐21 to AGO2 was significantly increased by TBK1^WT^, but not by TBK1^S172A^ only, TBK1^S172A^ and myr‐AKT (Figure S2D,E, Supporting Information). Taken together, above results demonstrate that TBK1 promotes miRNAs loading into AGO2 and miRISC formation in a kinase‐dependent manner.

### 2.3 TBK1 Phosphorylates AGO2 at S417 to Enhance the Formation and Activity of miRISC

**Figure 3:**
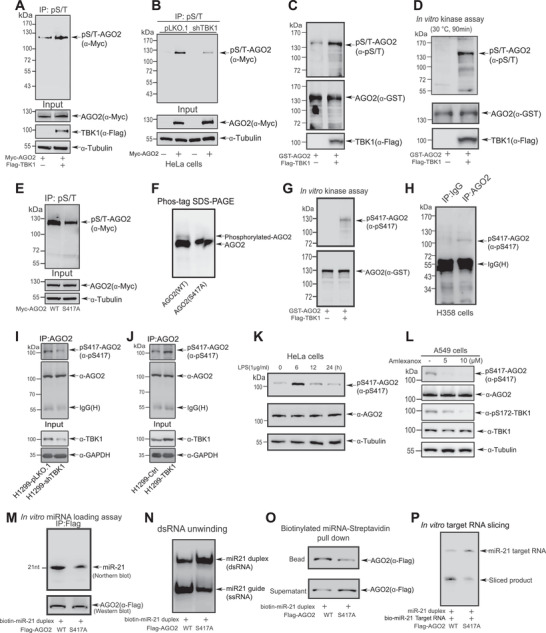
TBK1 phosphorylates AGO2 at S417 to enhance the formation and activity of miRISC. A) 293T cells transfected with Myc‐AGO2 with or without Flag‐TBK1 were lysed for IP with anti‐phospho‐Ser/Thr antibody, and followed by Western blotting analysis with anti‐Myc antibody. B) Lysates from HeLa‐shTBK1 or ‐pLKO.1 stable cells were used for IP with anti‐phospho‐Ser/Thr antibody, and followed by Western blotting analysis with anti‐Myc antibody. C) Purified GST‐AGO2 protein was incubated in lysates from 293T cells expressing Flag‐TBK1 or the control vector. The phosphorylation of GST‐AGO2 was analyzed by GST pull‐down and followed by Western blotting analysis with anti‐phospho‐Ser/Thr antibody. D) Purified GST‐AGO2 and Flag‐TBK1 were co‐incubated in the kinase reaction buffer containing ATP, subsequently the phosphorylation of GST‐AGO2 was analyzed by GST pull‐down and followed by Western blotting analysis with anti‐phospho‐S/T antibody. E) 293T cells transfected with Myc‐AGO2^WT^ or Myc‐AGO2^S417A^ were lysed for IP with anti‐phospho‐S/T antibody, and followed by Western blotting analysis with anti‐Myc antibody. F) Phos‐tag SDS‐PAGE showing the phosphorylation level of Myc‐AGO2^WT^ or Myc‐AGO2^S417A^ in 293T cells co‐transfected with TBK1. G) Purified GST‐AGO2 and Flag‐TBK1 were co‐incubated in the kinase reaction buffer containing ATP, subsequently the phosphorylation of GST‐AGO2 was analyzed by GST pull‐down and followed by Western blotting analysis with specific pS417‐AGO2 antibody. H–J) Lysates from H358 cells (H), stable cells H1299‐shTBK1 or ‐pLKO.1 I), and H1299‐TBK1 or ‐Ctrl () were used for IP with anti‐AGO2 antibody, and then immunoblotted by specific pS417‐AGO2 antibody. K) HeLa cells treated with LPS (1 µg ml^−1^) for the indicated times were lysed for Western blotting analysis by using the specific pS417‐AGO2 antibody. L) A549 cells treated with Amlexanox for 12 h before harvested. Lysates were used for Western blotting analysis with the indicated antibodies. M) Lysates from 293T cells transfected with Flag‐AGO2^WT^ or Flag‐AGO2^S417A^ were immunoprecipitated by Flag‐AGO2, then the beads were co‐incubated with biotin‐tagged miR‐21 duplex for in vitro miRNA loading assay. The miR‐21 recruited to AGO2 was detected by Northern blotting analysis. N) Flag‐AGO2 or Flag‐AGO2^S417A^ purified from 293T cells using 3×Flag peptide was incubated with miR‐21 duplex mimics for miR‐21 unwinding assays. The single‐stranded (ss) RNA molecules unwinding from double‐stranded (ds) miR‐21 substrates was detected on native polyacrylamide gels. O) Lysates from 293T cells transfected with Flag‐AGO2^WT^ or Flag‐AGO2^S417A^ were incubated with streptavidin‐Dynabeads‐coupled‐biotinylated miR‐21 duplex for biotinylated RNA‐streptavidin pull down assay. AGO2 pulled down by miR‐21 on the beads and not pulled down by miR‐21 were examined by Western blotting analysis with anti‐Flag antibody. P) Lysates from 293T cells transfected with Flag‐AGO2^WT^ or Flag‐AGO2^S417A^ were immunoprecipitated by Flag‐AGO2, and then were co‐incubated with miR‐21 duplex and biotin‐tagged miR‐21 target for in vitro target RNA slicing assay. The cleavage products were detected by Northern blotting analysis.

Since TBK1 directly interacted with AGO2, we speculated that AGO2 is a substrate of TBK1. To verify this, several biochemistry experiments were conducted. 293T cells transfected with Myc‐AGO2 with or without Flag‐TBK1 were lysed for IP with anti‐phospho‐Ser/Thr (pS/T) antibody, and followed by WB with anti‐Myc antibody, showing that overexpression of TBK1 increased the pS/T level of AGO2 compared with the vector (**Figure** **3**A). On the contrary, knockdown of TBK1 decreased the pS/T level of AGO2 in HeLa cells (Figure 3B). Moreover, bacterially expressed GST‐AGO2 protein was added to lysates from 293T cells ectopically expressing TBK1 or the vector for the incubation overnight, subsequently pulled down by GST‐beads for immunoblotting with anti‐pS/T antibody, showing that GST‐AGO2 protein in incubation with lysates from 293T cells overexpressing TBK1 was phosphorylated much higher than that from cells transfected the control vector (Figure 3C). An in vitro kinase assay was also carried out, in which bacterially expressed GST‐AGO2 and purified Flag‐TBK1 from 293T cells were co‐incubated in the kinase buffer for reaction, subsequently pulled down by GST beads and immunoblotted by anti‐pS/T antibody. The result showed that TBK1 was capable of catalyzing AGO2 phosphorylation in vitro (Figure 3D). Above results demonstrated that TBK1 is a new kinase for AGO2.

In order to identify TBK1‐dependent phosphorylation site of AGO2, first we used the PhosphoSitePlus program to predict that the sequence QPPS^417^*ILY of AGO2 protein is a highly conserved motif of TBK1.^[^ ^46^ ^]^ We have confirmed that S417 was indeed phosphorylated by mass spectrometry analysis (Figure S3A, Supporting Information). 293T cells transfected with Myc‐AGO2^WT^ or the point mutant Myc‐AGO2^S417A^ were lysed for IP with anti‐pS/T antibody, and followed by immunoblotting analysis with anti‐Myc antibody, showing that the pS/T level of AGO2^S417A^ was greatly reduced compared to that of AGO2^WT^ (Figure 3E)_,_ suggesting that S417 is a phosphorylation site of AGO2. The Phos‐tag SDS‐PAGE analysis further confirmed the S417 is a major phosphorylation site of AGO2 (Figure 3F).

To further confirm pS417 of AGO2 truly occurs in vivo, we generated a homemade antibody specifically against pS417 (anti‐pS417). To characterize the specificity of the antibody, we performed the dot‐blot assay to find that the anti‐pS417 antibody preferentially detected the phosphorylated peptide other than the unmodified peptide (Figure S3B, Supporting Information). The same in vitro kinase assay as Figure 3D was performed showing that TBK1 catalyzed pS417‐AGO2 (Figure 3G). By using this homemade AGO2 specific anti‐pS417 antibody, lysates from H358 cells were used for IP with anti‐AGO2 antibody and then immunoblotted by anti‐pS417 antibody, showing that endogenous pS417‐AGO2 could be detected (Figure 3H). We also verified that the level of pS417‐AGO2 was significantly decreased by stable knockdown of TBK1 in H1299 (H1299‐shTBK1) cells (Figure 3I) whereas it was increased by ectopically expressing TBK1 in H1299 (H1299‐TBK1) cells (Figure 3J). Further, we detected the levels of pS417‐AGO2 in different cell types treated with Lipopolysaccharides (LPS) for indicated time and found that the levels of pS417‐AGO2 were induced in various cell types after LPS stimulation (Figure 3K; Figure S3C–F, Supporting Information). On the contrary, the levels of pS417‐AGO2 were reduced in A549 (Figure 3L), H1975 (Figure S3G, Supporting Information) and H1299 cells (Figure S3H, Supporting Information) by Amlexanox, which is a specific inhibitor of TBK1^[^ ^47^ ^]^ and used to treat recurrent aphthous ulcers.^[^ ^48^ ^]^ Taken together, we proved TBK1 specifically phosphorylated AGO2 at S417 in vitro and in vivo.

To identify whether pS417‐AGO2 takes participate in the formation and activity of miRISC, we performed four similar experiments as in Figure 2. The in vitro miRNA loading assay showed that the guide strand of miR‐21 loading to AGO2^S417A^ was attenuated compared to that of AGO2^WT^ (Figure 3M). The dsRNA unwinding assay displayed that the formation of miR21‐guide (ssRNA) molecules from miR‐21‐duplex (dsRNA) was attenuated with AGO2^S417A^, compared to that of AGO2^WT^ (Figure 3N). The biotinylated miRNA‐Streptavidin pull‐down assay showed that AGO2^S417A^ (in beads) recruited to miR‐21 mimics was significantly decreased compared with AGO2^WT^ (Figure 3O). Finally, the in vitro target RNA slicing proved that more cleavage products were observed in AGO2^WT^ group than that in AGO2^S417A^group (Figure 3P). Collectively, above results demonstrated that TBK1 phosphorylates AGO2 at S417 to enhance the formation and activity of miRISC.

### 2.4 pS417‐AGO2 Promotes NSCLC Progression

**Figure 4:**
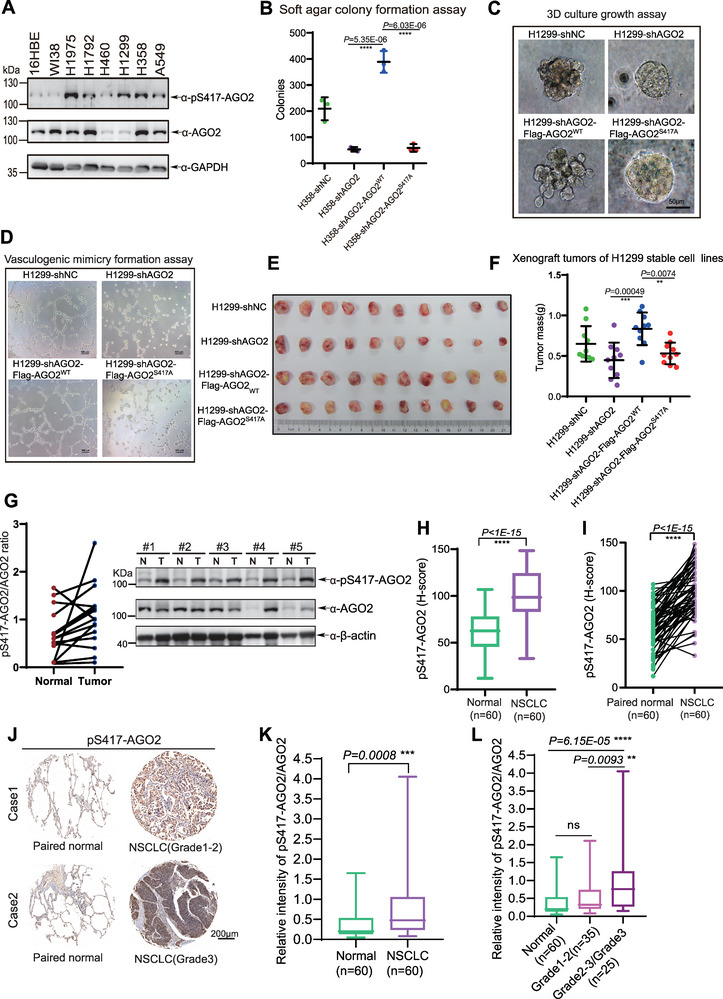
Phosphorylation of AGO2 at S417 promotes NSCLC progression. A) The phosphorylation levels of AGO2 in normal human bronchial epithelial cell line 16HBE, normal human lung fibroblasts cell line WI38, and NSCLC cell lines H1975, H1972, H460, H1299, H358 and A549 were analyzed by Western blotting analysis with specific anti‐pS417‐AGO2 and anti‐AGO2 antibodies. B) Soft agar colony formation assay for H358 stable cell lines was performed according to Methods. Data were presented as mean ± SD, n = 3. Statistical analysis was performed using one‐way ANOVA. ****p < 0.0001. C,D) 3D culture growth and vasculogenic mimicry (VM) for H1299 stable cell lines. Representative images of cell morphology C) and vasculogenic networks D) in extracellular matrix were taken. E,F) H1299 stable cell lines were subcutaneously injected into 6‐week‐old BALB/c nude mice individually. Mice were killed after 5 weeks of injection. Tumors were dissected E), and weight was assessed F). Data were presented as mean ± SD, n = 10. Statistical analysis was performed using one‐way ANOVA. **p < 0.01 and ***p < 0.001. (G) The phosphorylation levels of AGO2 in lung adenocarcinoma specimens and paired adjacent normal tissues were analyzed by Western blotting analysis with the specific pS417‐AGO2 antibody. Quantitative analysis for the ratio of pS417‐AGO2/AGO2 (left panel) (n = 17) and representative Western blotting analysis for pS417‐AGO2 levels (right panel). H‐J) IHC detection for pS417‐AGO2 in lung cancer tissue arrays. (H‐I) IHC staining scores for pS417‐AGO2 levels in paired normal tissues (n = 60) and NSCLC tissues (n = 60) were analyzed. Statistical analysis was performed using paired two‐tailed t‐test. ****p *< 0.0001*. J) Representative images of IHC staining for pS417‐AGO2 levels in normal tissues and pathological sub‐stages of NSCLC tissues. K) pS417‐AGO2/AGO2 ratio in normal tissues (n = 60) and NSCLC tissues (n = 60) were analyzed. Statistical analysis was performed using paired two‐tailed t‐test. ***p *< 0.001*. (L) pS417‐AGO2/AGO2 ratio in normal tissues (n = 60) and pathological sub‐stages of NSCLC tissues (Grade1‐2, n = 35; Grade2‐3/Grade2, n = 25) were analyzed. In box plots, the lines represent the median, first and third quartiles, the whiskers denote the minima and maxima. Statistical analysis was performed using one‐way ANOVA. **p < 0.01 and ****p *< 0.0001*.

To explore the biological function of pS417‐AGO2, we first examined the level of pS417‐AGO2 in various lung cancer cell lines with the specific anti‐pS417 antibody. Among them, WI38/VA13 is a normal human lung fibroblast cell line, 16HBE is a normal human bronchial epithelial cell line, and the rest are NSCLC cell lines. The results showed that the level of pS417‐AGO2 in NSCLC cell lines was much higher than that in normal cell lines (**Figure** **4**A). We generated stably re‐expressing AGO2^WT^ or AGO2^S417A^ in H358‐shAGO2 and H1299‐shAGO2, in which endogenous AGO2 was silenced by using the lentiviral shRNA system (Figure S4A,B, Supporting Information). To explore whether pS417‐AGO2 affects cell transforming potential, we performed a soft agar colony‐forming assay of stable H358 cell lines. The results showed that silencing of endogenous AGO2 in H358 cells decreased anchorage‐independent colony formation and growth, which was rescued by re‐expression of AGO2^WT^ but not mutant AGO2^S417A^ (Figure 4B). The 3D culture assay showed that knockdown of AGO2 in H1299 cells abolished cell penetrating into the matrigel, but instead, growing into tight colonies. Re‐expressed AGO2^WT^ cells proliferated diffusely and exhibited dispersed morphology, reflecting the great ability to invade the extracellular matrix. In contrast, AGO2^S417A^ cells grew into compact and round colonies (Figure 4C). Consistent with these results, the formation of vasculogenic mimicry (VM) was enhanced by ectopic expression of AGO2^WT^ but not AGO2^S417A^ (Figure 4D). To further investigate whether pS417‐AGO2 also influences tumor growth in vivo, above stable H1299 cell lines were subcutaneously inoculated into the backs of nude mice for the xenograft tumor growth assay. The photographs (Figure 4E) were taken and weight of tumors (Figure 4F) was measured, showing that the size and average weights of tumors in the AGO2^S417A^ group were reduced compared to those of in the AGO2^WT^. We have validated that the level of pS417‐AGO2 in AGO2^WT^ xenograft tumors was much higher than that of in AGO2^S417A^ xenograft tumors (Figure S4C, Supporting Information).

Next, we investigated the levels of pS417‐AGO2 in NSCLC specimens and their paired adjacent normal tissues by WB, showing that the levels of pS417‐AGO2 were significantly higher in tumors than those in adjacent normal tissues (Figure 4G and Table S1, Supporting Information). To further support this, we performed immumo‐histochemical staining (IHC) with pS417‐AGO2 antibody to detect the levels of pS417‐AGO2 in NSCLC tissue arrays (Table S2, Supporting Information) and Lung cancer tissue arrays (Table S3, Supporting Information), showing that the levels of pS417‐AGO2 were significantly higher in tumors than those in normal tissues (Figure 4H–J; Figure S4D, Supporting Information). We also found that the levels of pS417‐AGO2 were different in the pathological sub‐stage‐groups (Figure S4E,F, Supporting Information). Importantly, the ratio of pS417‐AGO2/AGO2 was significantly elevated in NSCLC samples (Figure 4K) and was positively correlated with tumor grade (Figure 4L). In contrast to the observed alterations in pS417‐AGO2 levels, the overall level of AGO2 protein remained relatively stable, indicating no significant changes between NSCLC and normal tissues (Figure S4G, Supporting Information). Of the samples analyzed, 83.33% of NSCLC specimens exhibited an increase in pS417‐AGO2 levels, with a decrease observed in 5% of the cases. On the contrary, at the levels of AGO2 protein, 33.33% of NSCLC samples showed an increase while 53.33% of samples showed a decrease (Figure S4H, Supporting Information). Moreover, analyses of AGO2 protein levels in the public database of Clinical Proteomic Tumor Analysis Consortium (CPTAC) revealed a decrease in both lung adenocarcinoma (n = 111) and lung squamous cell carcinoma samples (n = 110) (Figure S4I–K, Supporting Information). So, we concluded that the increase in pS417‐AGO2 levels in NSCLC was not attributed to the increase in AGO2 protein itself. Taken together, above findings demonstrate that pS417‐AGO2 promotes NSCLC development and the high pS417‐AGO2 levels are positively correlated with NSCLC progression, indicating that high pS417‐AGO2 level is a risk factor of NSCLC, and it may be a potential therapeutic target.

### 2.5 pS417‐AGO2 Promotes the Loadings of High‐Abundance Oncogenic miRNAs into AGO2 in NSCLC

**Figure 5:**
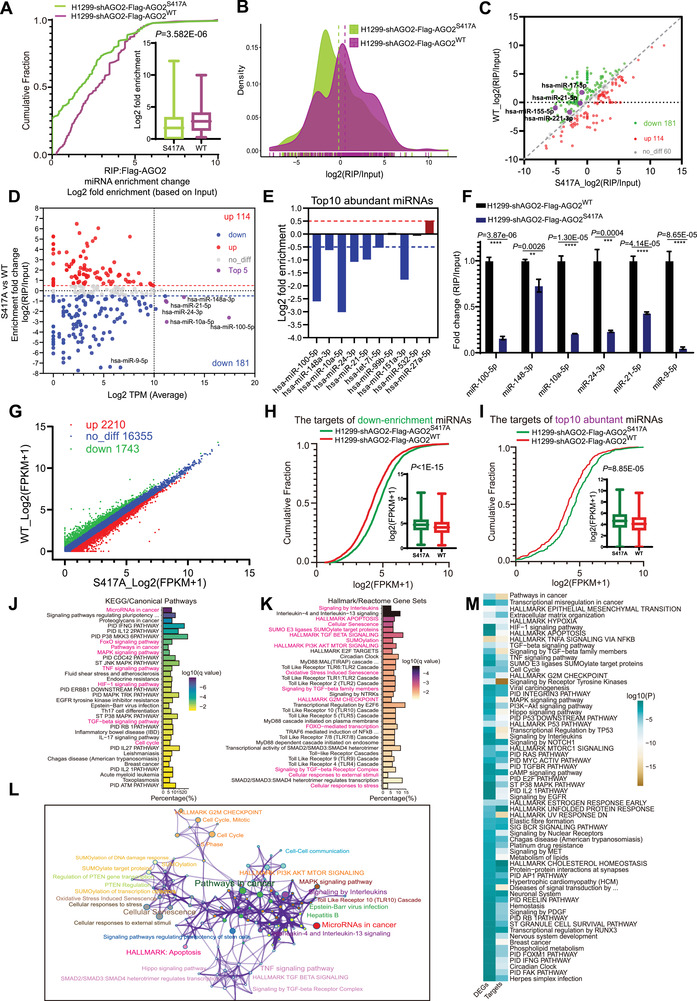
pS417‐AGO2 promotes the loadings of high‐abundance oncogenic miRNAs into AGO2 in NSCLC. A–C) Cumulative fraction analysis A), density distribution map B) and scatter plot C) of RIP‐Seq for miRNAs bound to AGO2 in stable cells H1299‐shAGO2‐Flag‐AGO2^WT^ and H1299‐shAGO2‐Flag‐AGO2^S417A^. *P*‐value was calculated using a two‐sided Mann‐Whitney U test for cumulative fraction analysis, n = 172; In box plots, the lines represent the median, first and third quartiles, the whiskers denote the minima and maxima. D) Scatterplot showing the fold change enrichment and abundance of miRNAs bound to AGO2. Significantly up‐enrichment (red), significantly down‐enrichment (blue) and top 5 most abundant (purple) miRNAs were indicated. E) Histograms showing the log2 fold change in the enrichment of top 10 most abundant miRNAs. F) Most abundant miRNAs including miR‐100‐5p, miR‐148‐3p, miR‐10a‐5p, miR‐24‐3p, miR‐21‐5p, miR‐9‐5p bound to AGO2 were examined by RIP‐qPCR. The enrichment of miRNAs associated with AGO2 was normalized by Input abundance of miRNAs. Data were presented as mean ± SD, n = 3. Statistical analysis was performed using unpaired two‐sided t‐test. **p < 0.01, ***p < 0.001 and ****p < 0.0001. G) Scatter plot showing the differentially expressed genes (DEGs) with log2 [fold change] ≥ 0.5 (up‐regulated) or log2 [fold change] < 0.5 (down‐regulated). H‐I) Cumulative fraction analyses for the abundance of targets of down‐enrichment miRNAs in RNA‐Seq, n = 2719 H), and the abundance of targets(of top 10 most abundant miRNAs in RNA‐Seq, n = 332 I). *P*‐values were calculated using a two‐sided Mann–Whitney U test; In box plots, the lines represent the median, first and third quartiles, the whiskers denote the minima and maxima. J) KEGG/Canonical Pathways enrichment analysis for targets of the top 10 most abundant miRNAs. K) Hallmark/Reactome Gene sets analysis for targets of the top 10 most abundant miRNAs. L) Representation of the most enriched pathways within targets of the top 10 most abundant miRNAs. M) Heatmap showing the top enrichment pathways by targets of the top 10 most abundant miRNAs and DEGs together, one row per cluster, using a discrete color scale to represent statistical significance.

In order to explore the global impact of pS417‐AGO2 on the miRNA pathway, we performed three high‐through sequences including miRNA‐Seq, AGO2‐RIP‐Seq, and mRNA‐Seq on above stable cells H1299‐shAGO2‐Flag‐AGO2^WT^ and H1299‐shAGO2‐Flag‐AGO2^S417A^. Based on the miRNA‐Seq, the miRNA profiles were analyzed to display that a few of miRNAs were changed between AGO2^WT^ and AGO2^S417A^ (Figure S5A, Supporting Information). The AGO2‐RIP‐Seq results showed that miRNAs associated with AGO2^S417A^ were significantly decreased compared to those with AGO2^WT^ by analyses of cumulative distribution curve (**Figure** **5**A) and density distribution diagram (Figure 5B). We divided miRNAs into the low affinity group (RIP down‐enrichment, log2 [fold change] < 0.5, RIP/input≥1) and the high affinity group (RIP up‐enrichment, log2 [fold change] ≥ 0.5, RIP/input≥1) according to the binding capability with AGO2^S417A^ versus AGO2^WT^. Distribution plot analysis showed that miRNAs in the low affinity group were more than that in the high affinity group (Figure 5C). pS417‐AGO2 may be required for the loading of critical miRNAs involved in the regulation of cell proliferation, drug‐resistance, immune response and autophagy, such as miRNA‐21,^[^ ^49^ ^]^ miR‐155,^[^ ^50^, ^51^ ^]^ miR‐17^[^ ^52^ ^]^ and miR‐221^[^ ^53^ ^]^ (Figure 5C). By combination analysis of above AGO2‐RIP‐Seq and miRNA‐Seq data, we found that the proportion of top 10 abundant miRNAs in H1299 cells was ≈92% (Figure S5B, Supporting Information). The five most abundant miRNAs, including miR‐100‐5p, miR‐148‐3p, miR‐10a‐5p, miR‐24‐3p, and miR‐21‐5p, belong to the low affinity group (Figure 5D), and most importantly, they are oncogenic miRNAs. Moreover, the binding affinity of the top 10 most abundant miRNAs to AGO2^S417A^ was significantly decreased compared to that of AGO2^WT^ (Figure 5E). To further confirm this, RIP‐qRT‐PCR was performed to show that the most abundant endogenous miRNAs, including miR‐100‐5p, miR‐148‐3p, miR‐10a‐5p, miR‐24‐3p, miR‐21‐5p, and miR‐9‐5p, had significantly lower binding levels with AGO2^S417A^ than with AGO2^WT^ (Figure 5F).

To define gene expression post‐transcriptionally regulated by pS417‐AGO2, differentially expressed genes (DEGs) of mRNA‐Seq were screened out according to the criteria of log2 [fold change] ≥ 0.5 (up‐regulated) or log2 [fold change] < 0.5 (down‐regulated). We identified 2210 up‐regulated and 1743 down‐regulated transcripts in AGO2^S417A^ group compared to that in AGO2^WT^ group (Figure 5G; Figure S5C, Supporting Information). We further explored the effect of pS417‐AGO2 on target mRNA levels. miRNA targets simultaneously predicted by three bioinformatic tools TargetScan, miRTarBase and miRDB were selected for the cumulative fraction analysis, showing that targets of down‐enrichment miRNAs in the AGO2^S417A^ group were more abundant than in the AGO2^WT^ group (Figure 5H). Similarly, the target mRNA levels of top 10 abundant miRNAs were also much higher in AGO2^S417A^ group than those in AGO2^WT^ group (Figure 5I). Above results reveal that pS417‐AGO2 promotes the loading of high‐abundance oncogenic miRNA into AGO2, facilitating target mRNA degradation and translational repression in NSCLC cells.

To further reveal the biological roles of miRNA targets, we performed functional enrichment analysis of KEGG/Canonical Pathways and Hallmark/Reactome Gene Sets to display that targets of top 10 miRNAs were enriched in cancer‐associated pathway such as pathway in cancer, cell cycle, cell apoptosis and so on (Figure 5J,K). Comprehensive enrichment analysis showed that targets of top 10 miRNAs were mainly involved in the regulation of tumorigenesis and development (Figure 5L). Furthermore, DEGs between AGO2^S417A^ and AGO2^WT^ group were also subject to the functional enrichment analysis of KEGG/Canonical Pathways and Hallmark/Reactome Gene Sets, showing that DEGs were also enriched in cancer‐associated pathway such as pathway in cancer, cell‐extracellular matrix (ECM) interaction, cell cycle (Figure S5D,E, Supporting Information). Moreover, Gene Ontology (GO) analysis showed that DEGs were enriched in the distinct gene clusters such as extracellular matrix binding, regulation of cell adhesion and so on (Figure S5F,G, Supporting Information). Comprehensive enrichment analysis showed that DEGs regulated by pS417‐AGO2 were also involved in the pathway in cancer (Figure S5H, Supporting Information), which was highly consistent with the functional properties of top 10 miRNA targets (Figure 5L).

To evaluate the crossover between top 10 miRNA targets and DEGs regulated by pS417‐AGO2, we performed an integrative analysis of miRNA targets and DEGs. The circos plot showed a partial overlap between top 10 miRNA targets and DEGs (Figure S5I, Supporting Information), suggesting a convergence of pathways in the two datasets. For a comprehensive comparison of significant pathways, we performed pathway enrichment analysis of miRNA targets and DEGs for meta‐analysis using Metascape. The results indicated a substantial overlap in the enriched pathways between miRNA targets and DEGs (Figure 5M). Taken together, these results demonstrate that p417‐AGO2 regulates gene expression profiles mainly by increasing miRISC formation.

### 2.6 Combining Antagonism of Oncogenic miRNAs and Reduction of Oncogenic miRISC Formation for the Treatment of NSCLC

**Figure 6:**
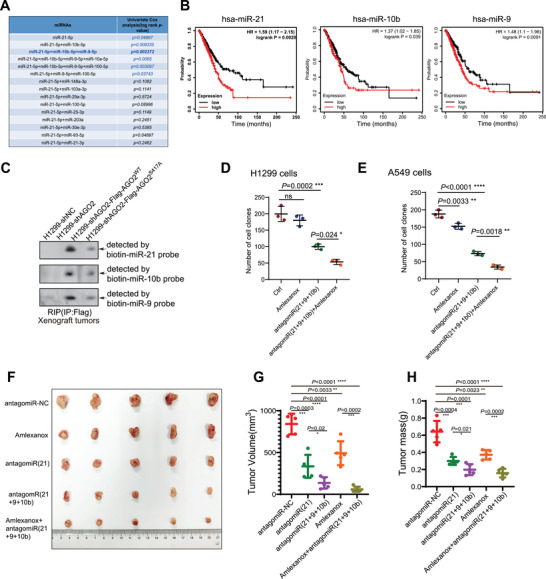
Combining antagonism of oncogenic miRNAs and reduction of oncogenic miRISC formation for the treatment of NSCLC. A) The optimal miRNA combination of miRNA‐based therapeutic strategies for NSCLC patients was developed by univariate cox analysis. B) The correlations between the expression levels of miRNAs (miR‐21, miR‐10b, miR‐9) and the survival of lung adenocarcinoma(LUAD) patients were analyzed by the Kaplan‐Meier analysis and and compared by the log‐rank test. C) The miR‐21, miR‐9, and miR‐10b loading in xenografted tumors were detected by RIP‐Northern blotting analysis. D,E) Colony formation assays of H1299 D) and A549 E) cells. Cells were grown in the absence or presence of the indicated only antagomiRs, Amlexanox, or combinations for 10–12 days, stained and the number of colonies was scored. Data were presented as mean ± SD, n = 3. Statistical analysis was performed using one‐way ANOVA. *p < 0.05, **p < 0.01, ***p < 0.001 and ****p < 0.0001. F–H) Mice were subcutaneously injected with 1 × 10^6^ H1299 cells. Once tumors reached an average of 5 mm × 5 mm (14 days), the mice were treated with antagomiRs (5 nmol) by intratumoral injection and TBK1 inhibitor Amlexanox (25 mg k^−1^g) by intraperitoneal injection for twice per week. 28 days after inoculation, xenograft tumors were dissected F), tumor volume G) and weight H) were assessed. Data were presented as mean ± SD, n = 5 in G) and H). Statistical analysis was performed using unpaired two‐sided t‐test. *p < 0.05, **p < 0.01, ***p < 0.001 and ****p < 0.0001.

We found that the loading of highly abundant oncogenic miRNAs into AGO2 to form miRISC was increased in NSCLC, suggesting that intervening or reducing these oncogenic miRISC formation could be a promising antitumor therapeutic strategy. Identifying the optimal miRNA candidates or targets for each disease type remains a significant challenge in the development of miRNA‐based therapeutics. By combining with our RIP‐Seq data, we further analyzed the miRNA‐Seq data of 30 tumor types in the TCGA (The Cancer Genome Atlas) database to find the high‐abundance miRNAs (Figure S6A, Supporting Information). To develop a novel miRNA‐based therapeutic strategy for NSCLC patients, we screened the optimal miRNA combination through univariate cox analysis. Many studies have shown that miR‐21 has oncogenic effects and is significantly upregulated in tumors compared to normal tissues. Therefore, we conducted multi‐target combination analysis using a set of high‐abundance miRNAs centered around miR‐21. The univariate cox analysis revealed that the combined treatment of miR‐21, miR‐9, and miR‐10b was the most effective therapeutic strategy (**Figure** **6**A; Figure S6B, Supporting Information). By analyzing public clinical data, we found that the expression levels of miR‐21, miR‐9, and miR‐10b in a clinical large sample of NSCLC cases were significantly higher than those in paired normal tissues (GEO database GSE137140) (Figure S6C, Supporting Information), and the Kaplan‐Meier survival analysis revealed that patients with the high levels of miR‐21, miR‐9, or miR‐10b had a lower survival rate in a variety of tumors (Figure 6B; Figure S6D, Supporting Information). We also detected miR‐21, miR‐9, and miR‐10b loading in xenografted tumors (Figure 4E) by RIP‐Northern blotting analysis, showing that the loadings of these three miRNAs in xenografted tumors of AGO2^WT^ group were all higher than that of in AGO2^S417A^ group (Figure 6C), suggesting that their functions were regulated by pS417‐AGO2.

To explore the role of selected miRNAs in NSCLC, antagomiRs that are synthetic antagonists of miRNAs^[^ ^54^ ^]^ were utilized for the colony formation assa*y*. By using three types of antagomiRs to inhibit miR‐21, miR‐9 and miR‐10b, respectively, the colony number was significantly reduced, while the addition of the TBK1 inhibitor Amlexanox further enhanced the inhibitory in H1299 and A549 cells (Figure 6D,E). This result suggests that, in addition to the three antagomiRs, Amlexanox also interferes with oncogenic miRISC formation by inhibiting pS417‐AGO2 levels, thereby exerting an enhanced synergistic inhibitory effect.

To further access the in vivo therapeutic effect of above strategy, H1299 cells were subcutaneously injected into nude mice. Once tumors reached an average of 5 mm × 5 mm in size, 5 nmol of antagomiR(NC), antagomiR(21), or antagomiRs(21+9+10b) in 50 µl saline buffer were locally injected into the tumors for twice per week. To avoid the influence of dose effect, the dosage of each component in the antagomiRs(21+9+10b) mixture was 1.67 nmol, and the total dosage of the antagomiRs(21+9+10b) mixture was the same as that of antagomiR(21) or antagomiR(NC). Compared with antagomiR(NC), the treatment of antagomiR(21) greatly reduced tumor sizes (Figure 6F,G) and weights (Figure 6H). More importantly, the inhibition of tumor growth by antagomiRs(21+9+10b) was more obvious than that of antagomiR (21), suggesting that multi‐target strategy against oncogenic miRNAs is superior to the single target strategy. Moreover, to investigate the combined therapeutic effect of antagomiRs and Amlexanox in vivo, mice were treated with Amlexanox (25 mg k^−1^ g) by intraperitoneal injection for twice per week. In contrast to only Amlexanox treatment, the combination treatment of Amlexanox and antagomiRs(21+9+10b) showed the maximum inhibitory effect on tumor growth (Figure 6F–H). These results reveal that the therapeutic strategy combining antagonism of oncogenic miRNAs and Amlexanox reduction of oncogenic miRISC formation provides an effective approach for the development of miRNA‐based therapy for NSCLC.

### 2.7 Gefitinib Combined with Amlexanox for the Treatment of Drug‐Resistant NSCLC

**Figure 7:**
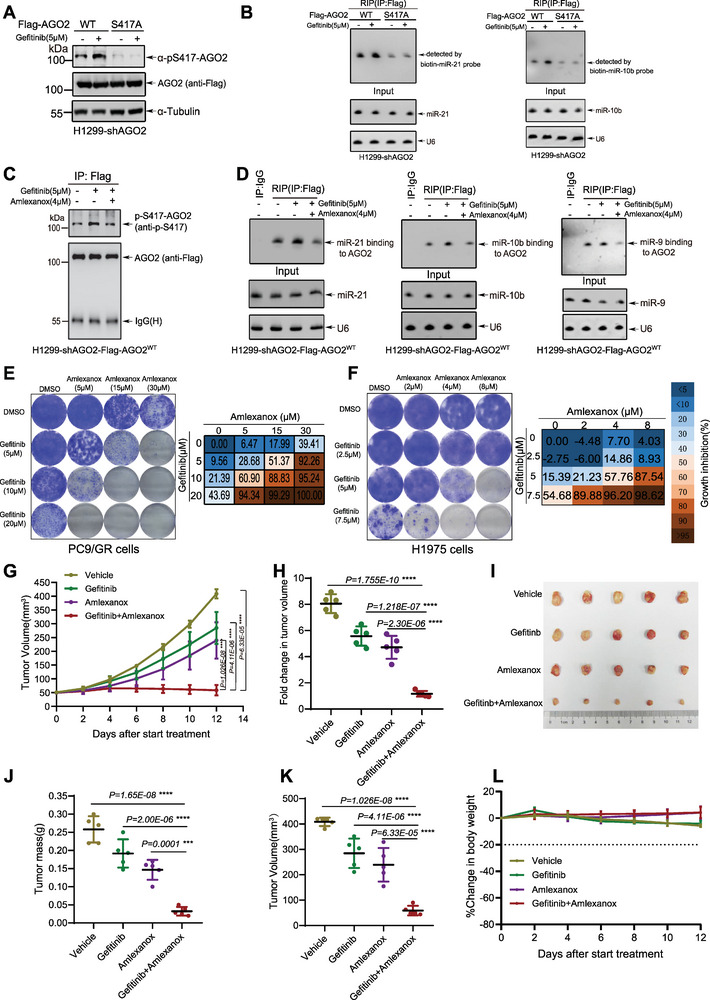
Gefitinib combined with Amlexanox for the treatment of drug‐resistant NSCLC. A) H1299‐shAGO2‐Flag‐AGO2_WT_ _or_^S417A^ cells were treated with Gefitinib (5 µM) and together with or without Amlexanox (4 µM) simultaneously for 12 h before harvested. Cells were lysed for IP with anti‐Flag antibody, and then immunoblotted by specific anti‐pS417‐AGO2 antibody. B) H1299‐shAGO2‐Flag‐AGO2_WT_ _or_^S417A^ cells treated with or without Gefitinib (5 µM) were lysed for the RIP assay with anti‐Flag antibody, and then miR‐21, miR‐10b associated with AGO2 were detected by Northern blotting analysis. C) H1299‐shAGO2‐Flag‐AGO2^WT^ cells were treated with Gefitinib (5 µM) together with or without Amlexanox (4 µM) simultaneously for 12 h before harvested. Cells were lysed for IP with anti‐Flag antibody, and then immunoblotted by specific anti‐pS417‐AGO2 antibody. D) H1299‐shAGO2‐Flag‐AGO2^WT^ cells treated with Gefitinib (5 µM) together with or without Amlexanox (4 µM) simultaneously for 12 h before harvested. Cells were lysed for the RIP assay with anti‐Flag antibody, and then miR‐21, miR‐9, and miR‐10b associated with AGO2 were detected by Northern blotting analysis. E,F) PC9/GR E) and H1975 F) cells were treated with Gefitinib, Amlexanox or their combination at the indicated concentrations. Cells were fixed and stained after 10–12 days. Representative data from three independent experiments. G–L) Mice were subcutaneously injected with 4 × 10^6^ PC9/GR cells. Once tumors reached an average of 5 mm × 5 mm, the mice were treated with Gefitinb (20 mg k^−1^ g) by oral gavage, Amlexanox (20 mg k^−1^ g) by intraperitoneal injection, or combination therapy for every 2 days. The tumor growth curve during treatment period G) and fold change in tumor volume pre or post‐treatment was analysed H). Xenograft tumors were dissected I). Tumor mass J) and Tumor volume K) after treatment were assessed. L) The change of body weight during treatment period were assessed. Data were presented as mean ± SD, n = 5. Statistical analysis was performed using one‐way ANOVA. ***p < 0.001 and ****p < 0.0001.

Although EGFR tyrosine kinase inhibitors (TKIs) offer an effective treatment for patients with advanced NSCLC, therapeutic strategies to overcome acquired resistance to EGFR‐TKIs in NSCLC patients remains to be determined. Interestingly, we found that Gefitinib, the first selective TKI of EGFR, significantly increased (**Figure** **7**A) and the loadings of miR‐21 and miR‐10b (Figure 7B) to AGO2 in H1299‐shAGO2‐AGO2^WT^ but not in H1299‐shAGO2‐AGO2^S417A^ cells. More importantly, the Gefitinib‐induced pS417‐AGO2 was inhibited by the addition of Amlexanox in H1299‐Flag‐AGO2 cells (Figure 7C). Accordingly, the treatment of Gefitinib increased loadings of three oncogenic miRNAs miR‐21, miR‐10b and miR‐9 into AGO2, which were significantly decreased by the co‐treatment with Amlexanox (Figure 7D). The levels of pS417‐AGO2 varied significantly among different NSCLC cells (Figure S7A, Supporting Information). Notably, the result showed that the levels of pS417‐AGO2 in PC9 and HCC827 were significantly lower than those in PC9/GR (Gefitinib‐acquired Resistant), HCC827/GR (Gefitinib‐acquired Resistant) and other cells examined (Figure S7A, Supporting Information). PC9 and HCC827 cells are distinguished by the presence of the EGFR exon 19 deletion mutation, rendering them notably responsive to EGFR‐TKIs.^[^ ^55^ ^]^ PC9/GR and HCC827/GR cells were established from its parental sensitive cell lines PC9 and HCC827 after long time exposure to Gefitinib. H1299 and H1975 cells also were relatively insensitive to Gefitinib.^[^ ^56^ ^]^ Amlexanox treatment reduced the levels of pS417‐AGO2 in PC9/GR (Figure S7B, Supporting Information) and HCC827/GR cells (Figure S7C, Supporting Information). Compared with the parental PC9 and HCC827 cells, PC9/GR (Figure S7C Supporting Information) and HCC827/GR (Figure S7D Supporting Information) cells showed significantly stronger resistance to Gefitinib. Since Gefitinib enhanced the formation and activity of oncogenic miRISCs through TBK1‐mediated pS417‐AGO2, which might contribute to acquired resistance to Gefitinib, we speculated that the co‐treatment with Amlexanox may overcome the resistance to Gefitinib. Therefore, we compared the inhibitory effects of a single Amlexanox or Gefitinib, and a dual combination on the growth of PC9/GR, HCC827/GR, H1299 and H1975 cells. The combination of Amlexanox and Gefitinib significantly enhanced the inhibitory effect on the growth of PC9/GR (Figure 7E), H1975 (Figure 7F), HCC827/GR (Figure S7F, Supporting Information), and H1299 cells (Figure S7G, Supporting Information).

Next, we explored the combination of Gefitinib and Amlexanox as a therapy strategy to drug‐resistant NSCLC in vivo. We employed PC9/GR cells for in vivo experiments. We compared the treatment effects using a lower dose of Gefitinib (20 mg k^−1^ g) with/without Amlexanox (20 mg k^−1^ g) in established tumors from PC9/GR cells. PC9/GR cells were subcutaneously injected into nude mice. After two weeks, mice were treated with Gefitinb by oral gavage, with/without Amlexanox by intraperitoneal injection. The results showed that compared to single drug treatment with Gefitinib or Amlexanox, the dual combination therapy resulted in significant tumor regression (Figure 7G–K). Tumor growth arrest proved this (Figure 7G,H). Notably, the average sizes (Figure 7I,J) and weights (Figure 7K) of tumors in the combination treatment group exhibited a significant reduction compared to those in the vehicle group and the single drug groups. The combination treatment with Gefitinib and Amlexanox dramatically suppressed tumor growth without significant loss of body weight (Figure 7L). To further verify the results of in vivo experiments on PC9/GR cells, H1975*^luc^* cells were also used for in vivo experiments. Consistent with in vivo results of PC9/GR cells, the treatment with the dual combination therapy inhibited tumor growth more effectively compared with the treatment of Gefitinb (Figure S7H–L, Supporting Information) without a notable loss of body weight (Figure S7M, Supporting Information). Taken together, these results suggest that oncogenic miRNA loading partially contributes to EGFR‐TKI resistance, and the combination therapy with Amlexanox represents a particularly potent approach to overcome EGFR‐TKI resistance of NSCLC.

## 3 Discussion

AGO2 plays a central effector role in the miRNA pathway, undergoing post‐translational modifications (PTMs) at different locations, each of which has a specific impact on its function. Multiple evidences have shown that the ubiquitin‐proteasome system controls the stability and turnover of AGO protein.^[^ ^57^, ^58^, ^59^, ^60^ ^]^ Hydroxylation at proline 700 of AGO2 is important for AGO2 stability and programmed RISC activity.^[^ ^61^ ^]^ AGO proteins are modified by poly (ADP‐ribose) upon stress, which alleviates miRNA‐mediated translational repression and miRNA‐directed mRNA cleavage.^[^ ^62^ ^]^ Our previous study showed that acetylation of AGO2 occurring at three sites K720, K493, and K355 specifically promotes oncogenic miR‐19b biogenesis^[^ ^63^ ^]^ and Met1‐linked linear ubiquitination of of AGO2 impedes its recruitment of miRNA‐targeted mRNAs.^[^ ^37^ ^]^ Phosphorylation is the most common PTMs occurring at different residues of AGO2. Y529, located in the small RNA 5′‐end‐binding pocket of AGO2 protein can be phosphorylated (pY529‐AGO2), which prevents the effective binding of small RNA to AGO2.^[^ ^64^ ^]^ pY393‐AGO2 mediated by EGFR or c‐SRC reduces the binding of DICER to AGO2 and thereby inhibits miRNA processing from long‐loop precursor miRNAs to mature miRNAs in response to hypoxia stress.^[^ ^65^, ^66^ ^]^ Akt3‐mediated pS387‐AGO2 facilitates its interaction with GW182 and localization to cytoplasmic processing bodies (P bodies), which is a molecular switch between target mRNA cleavage and translational repression activities of AGO2.^[^ ^67^ ^]^ Phosphorylation of AGO2 at S824‐S834, located at a structurally unresolved loop of PIWI domain, impairs its interaction with target mRNA and subsequently suppresses miRNA‐mediated gene silencing.^[^ ^68^, ^69^ ^]^ TBK1 is known to play important roles in innate immune signaling^[^ ^70^ ^]^ and AKT/mTORC1 pathway activation for cancer cell survival.^[^ ^71^ ^]^ In this study, we identified that TBK1 catalyzed the phosphorylation of AGO2 at S417 with Gefitinib, which enhanced miRNA loading to AGO2 to form effective miRISC.

One of the biggest challenges in developing miRNA‐based therapeutics is to identify the best miRNA candidates. Numerous cancer studies have shown that miR‐21 has an oncogenic role and is significantly upregulated in tumors compared with normal tissues. A recent report analyzing lung adenocarcinoma sequencing data from TCGA demonstrates that the locus containing the miR‐21 gene is amplified, and that amplification in this genomic region acts as a prognostic marker.^[^ ^72^ ^]^ However, targeting miR‐21 alone is not effective in suppressing tumor cells in vivo.^[^ ^73^ ^]^ We used the univariate cox analysis to perform combined calculations on a series of high‐abundance miRNAs, and found the most effective therapeutic strategy that was the combination targeting of miR‐21, miR‐9, and miR‐10b. Indeed, compared with only targeting miR‐21 treatment, the combination targeting of miR‐21, miR‐9, and miR‐10b was more effective. More importantly, we found the antimiRs(21+9+10b) treatment combined with the TBK1 inhibitor Amlexanox, which suppressed the formation and activity of oncogenic miRISCs, had the best anti‐tumor effect (Figure 6).

Lung cancer is a malignant tumor with one of the highest morbidity and mortality rates.^[^ ^1^ ^]^ Active mutations in driver genes such as *EGFR* and *KRAS* are commonly present in patients and are key targets for anti‐tumor therapy. EGFR‐TKIs provide an effective treatment for NSCLC patients, however the treatment strategy for overcoming acquired resistance to EGFR‐TKIs in NSCLC patients remains to be determined. We found that Gefitinib induced pS417‐AGO2, thereby promoting the loadings of oncogenic miRNAs into AGO2 to form effective miRISCs, which was a potential mechanism for Gefitinib resistance in NSCLC cancer cells. The oncogenic miRNAs contribute to acquired resistance to Gefitinib, such as miR‐21,^[^ ^49^ ^]^ miR221^[^ ^53^ ^]^ and so on. Based on this, we developed a strategy of combining Gefitinib with Amlexanox to intervene the formation and activity of oncogenic miRISCs, which significantly increased the sensitivity of Gefitinib to NSCLC cells (Figure 7).

Although some studies have reported that AGO2 overexpression may be correlated with lung cancer progression, there was no significant difference in overall levels of AGO2 protein between NSCLC and normal tissues according to our clinical data (Figure S4G,H, Supporting Information). Analyses on large‐scale NSCLC samples from the public database CPTAC also showed the levels of AGO2 protein were even downregulated (Figure S4I–K, Supporting Information). However, the levels of pS417‐AGO2 and the ratios of pS417‐AGO2/AGO2 in NSCLC samples were significantly higher than those in normal tissues. Thus, we concluded that the increase in pS417‐AGO2 levels in NSCLC was not attributed to the increase in AGO2 protein itself.

**Figure 8:**
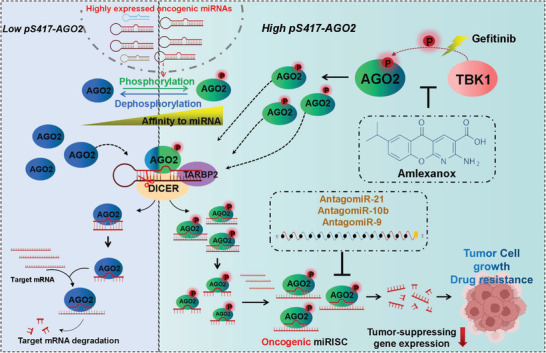
A model summarizing that TBK1‐mediated pS417‐AGO2 promotes NSCLC progression and resistance to Gefitinib by increasing the formation and activity of oncogenic miRISCs.

In summary, our study demonstrated that TBK1 is a novel kinase that phosphorylates AGO2 at S417 and can be induced by EGFR‐TKI Gefitinib. High level of pS417‐AGO2 promotes NSCLC progression and resistance to Gefitinib by increasing the formation and activity of oncogenic miRISCs (**Figure** **8**). The high level of pS417‐AGO2 in clinical specimens is positively correlated with poor prognosis in lung cancer patients and is one of the high‐risk factors for NSCLC, suggesting that the measurement of pS417‐AGO2 levels may become a new clinical diagnostic indicator. Most importantly, using the TBK1 inhibitor Amlexanox to reduce pS417‐AGO2 is a good strategy for treating NSCLC.

## 4 Experimental Section

### Cell Cultures

The human lung cancer cell lines NCI‐H1299, NCI‐H1975, NCI‐H1650, A549, HCC827 and HCC827/GR cells were cultured in RPMI 1640 (Hyclone, Logan, UT, USA) containing 10% fetal bovine serum (Biowest, Kansas, MO, USA), 1% penicillin and streptomycin (Invitrogen, CA, USA) at 37 °C with 5% CO_2._ The normal human lung fibroblasts cells WI38/VA13 were cultured in MEM medium supplemented with 10% FBS, 1% penicillin and streptomycin, 1% non‐essential amino acids, 1 mM sodium pyruvate at 37 °C with 5% CO_2._ HEK293T, HeLa, PC9, and PC9/GR cells were cultured in Dulbecco's modified Eagle's medium (DMEM, Hyclone) containing 10% FBS, 1% penicillin and streptomycin at 37 °C with 5% CO_2_. HCC827/GR and PC9/GR cells were from Professor Xiao's research group.^[^ ^74^ ^]^ Other cell lines are purchased from National Collection of Authenticated Cell Cultures.

### In Vitro Target RNA Slicing Assay

For the in vitro target RNA slicing assay, 293T cells transiently expressing Flag‐AGO2 were lysed in cell lysis buffer (50 mM Tris/HCl pH 7.4, 150 mM NaCl, 1% NP‐40, and Complete Protease Inhibitor Cocktail) for 1 h on ice. Lysates were centrifuged for 30 min at 4 °C after sonication, and then supernatants were transferred into new tubes and incubated with anti‐Flag M2 affinity beads overnight at 4 °C. The beads were washed three times with high‐salt lysis buffer containing 500 mM NaCl and Flag‐AGO2 were purified using the 3×Flag peptide according to the manufacturer's specifications (Sigma). miR‐21 mimic and linearized 5′end‐biotinylated miR‐21 target RNA were incubated with the purified Flag‐AGO2 protein at 37 °C for 4 h in a 10‐ml reaction volume. The reaction contained yeast tRNAs, 25 mM HEPES‐KOH pH 7.5, 50 mM potassium acetate, 5 mM magnesium acetate, 5 mM dithiothreitol (DTT), 1 µl of 293T cell lysates. The RNA was purified from the reaction mixture and analyzed on 20% urea polyacrylamide gel.

### In Vitro miRNA Loading Assay

For the in vitro miRNA duplex loading assay, 293T cells transfected with indicated plasmids were lysed with RIP‐lysis buffer (50 mM Tris‐HCl pH7.4, 150 mM NaCl, 10 mM EDTA, 5 mM MgCl_2_, 1% NP‐40, 1 mM DTT, 100 units ml^−1^ RNase inhibitor, 400 µM VRC and Protease inhibitor cocktail). Lysates were performed by IP with anti‐Myc or Flag (AGO2) antibody on proteinA/G agarose beads. The beads with AGO2 complex were incubated with 100 nM biotin tagged miR‐21/let‐7a duplex (biotin labeled at the guide strand) in a 20‐µl reaction buffer (20 mM Tris‐HCl pH 7.5, 200 mM KCl, 2 mM MgCl_2_, 5% glycerol, 1 mM DTT, 40 U RNase inhibitor) for 1 h at 37 °C, followed by washing of the beads five times with RIP‐lysis buffer. The RNA was purified from beads and analyzed on 20% urea polyacrylamide gel.

For the pre‐miRNA cleavage‐coupled miRISC loading assay, the biotin‐pre‐miR‐21 or pre‐let‐7s were transcribed and labeled using the ScriptMAXTM Thermo T7 Transcription Kit (TSK‐101; TOYOBO) and Biotin‐16‐UTP (#11 388 908 910; Roche, Indianapolis, IN, USA) according to the manufacturer's guidelines. The reaction mixture in 20 mM HEPES (pH 6.5), 1.5 mM MgCl_2_, 100 mM KCl, 80 mM NaCl, 1 mM DTT, 40 U RNase inhibitor, and 10% glycerol containing AGO2 complex‐coupled proteinA/G agarose beads. Subsequently, 0.5–1 µM pre‐miR‐21 or pre‐let‐7a‐3 with biotin labeling was added to the reactions and further incubated for dicing and loading of pre‐miR‐21 or pre‐let‐7a‐3 to AGO2/RISC at 37 °C for 30 min. The beads were washed five times with RIP‐lysis buffer, and the RNA was purified from the reaction mixture and analyzed on 20% urea polyacrylamide gel.

### Biotinylated miRNA‐Streptavidin Pull Down Assay

293T cells transfected with indicated plasmids were lysed with RIP‐lysis buffer (25 mM Tris/HCl pH 8.0, 150 mM NaCl, 2 mM MgCl_2_, 1% NP‐40, 1 mM DTT, Protease Inhibitor Cocktail, and 40U RNase inhibitor). 100 pmol of biotin‐miR‐21 duplex mimics were incubated with 50 µl of Dynabeads MyOne Streptavidin C1 (#65 001; Invitrogen) for 30 min, and then beads were washed with washing buffer (5 mM Tris/HCl pH 7.5, 0.5 mM EDTA, 1 M NaCl) for three times. The biotin‐miR‐21 duplex coupled Dynabeads were mixed with cell lysates, and then incubated overnight at 4 °C. Fractions unbound to the beads were obtained from supernatant. The Dynabeads were washed with RIP‐lysis buffer for three times. AGO2 on the beads or in the supernatant were examined by Western blotting analysis with indicated antibodies.

### miRISC Capture Assay

This protocol is very similar to the biotinylated miRNA‐streptavidin pull down assay. 293T cells transfected with indicated plasmids were lysed with RIP‐lysis buffer (25 mM Tris/HCl pH 8.0, 150 mM NaCl, 2 mM MgCl_2_, 0.5% NP‐40, 1 mM DTT, Protease Inhibitor Cocktail, and 40 U RNase inhibitor). 100 pmol of biotin‐miR‐21 target RNA were incubated with 50 µl of Dynabeads for 30 min, and then the beads were washed with washing buffer (5 mM Tris/HCl pH 7.5, 0.5 mM EDTA, 1 M NaCl) for three times. The biotin‐miR‐21 target RNA coupled Dynabeads were mixed with cell lysates and 10 pmol of miR‐21 duplex, and then incubated overnight at 4 °C. Fractions unbound to the beads were obtained from supernatant. The Dynabeads were washed with RIP‐lysis buffer for three times. miRISCs on the beads or in the supernatant were examined by Western blotting analysis with indicated antibodies.

### In Vitro miRNA Unwinding Assay

Cell extracts were prepared as the following protocol. Briefly, cells were washed 2 times with cold PBS and resuspended in hypotonic lysis buffer (10 mM HEPES‐KOH, pH 7.5, 10 mM potassium acetate, 0.5 mM magnesium acetate, 0.1% Tween‐20, EDTA‐free protease inhibitor cocktail). Collected cells were incubated at 4 °C for 30 min and centrifuged at 16 000 g for 10 min at 4 °C, and then the supernatant were quickly frozen and stored at 80 °C until use. For in vitro miRNA unwinding assay, 10 µl of reaction containing purified Flag‐AGO2, 250 fmol of miR‐21 duplex (biotin labeled at the guide strand), 1 µl of cell extracts (10 mg ml^−1^), 30 mM HEPES pH 7.4, 100 mM potassium acetate, 2 mM magnesium acetate, 5 mM DTT, protease inhibitor cocktail (Roche), 25 mM creatine phosphate, 1 mM ATP, and 0.5 U µl^−1^ creatine kinase incubated for 10 min at 25 °C. Then, proteinase K (2 mg mL^−1^) was added into the reaction and incubated at 25 °C for an additional 10 min. Samples were rapidly transferred to ice, mixed with gel loading buffer. The formation of single‐stranded (ss) RNA molecules from double‐stranded (ds) miR‐21 substrates were detected by native polyacrylamide gels.

### MiR‐21/let‐7a Luciferase Activity Reporter

Four‐repeated miR‐21binding site (BS) sequences of 4×miR‐21‐BS were inserted into the 3′‐UTR of Renilla luciferase of the vector psiCHECK2 to constitute psiCHECK2‐ 4×miR‐21‐BS. Accordingly, Renilla luciferase is expectedly attenuated when let‐7a binds to binding sites. AGO2, psiCHECK2‐4×miR‐21‐BS and indicated plasmids or miR‐21 were co‐transfected into 293T cells, 48 h after transfection cells were subjected to the dual‐luciferase reporter assay according to the manufacturer's instruction. The miRSIC activity with *Renilla* luciferase was normalized by Firefly luciferase

### RNA Immunoprecipitation‐Northern Blotting Assay (RIP‐NB Assay)

RNA immunoprecipitation assay (RIP) was performed as previously described.^[^ ^63^, ^75^ ^]^ Cells transfected with indicated plasmids were lysed with RIP lysis buffer [50 mM Tris‐HCl pH 7.4, 150 mM NaCl, 2 mM MgCl_2_, 1% NP40, 1 mM dithiothreitol, 100 U ml^−1^ RNase inhibitor (Fermentas), 400 µM VRC (New England BioLabs) and Protease inhibitor cocktail (Roche)] for 30 min on ice, then centrifuged at 16 000 g for 20 min to clear the lysate. One‐tenth of lysates was used as input, and other of lysates were incubated with protein A/G agarose beads and antibodies at 4 °C overnight. The beads were washed five times with RIP lysis buffer containing 300 mM NaCl and the bound RNAs was isolated using Trizol (Sigma) following instructions. The following step was performed according to the protocol of Northern blotting analysis.

### Northern Blotting Analysis

The Northern blotting analysis of RNA was conducted as described before.^[^ ^63^, ^75^ ^]^ Briefly, total RNAs were isolated with TRIzol reagent (Invitrogen) and denatured at 95 °C for 5 min. Then, RNAs were separated by electrophoresis on the 20% polyacrylamide 8 M urea gel and transferred to the nylon membrane (Roche). After cross‐linking, RNAs were incubated with the specified biotin probes and detected by using Chemiluminescent Nucleic Acid Detection Module kit (Thermo Scientific) following instructions.

### Quantitation of AGO2‐Bound miRNAs

AGO2‐bound miRNAs were quantitated by qRT‐PCR. Briefly, RNAs from immunoprecipitated AGO2 and lysates were extracted using Trizol, treated with DNase, and cDNAs were generated. The cDNA library was then used as a template for qRT‐PCR using a SYBR Green master mix(Applied Biosystems). The enrichment of miRNAs associated with AGO2 was normalized by input abundance of miRNAs.

### Soft‐Agar Colony Formation Assay

The ability of anchorage‐independent growth was evaluated by a soft agar assay as described.^[^ ^76^ ^]^ Briefly, 2 × 10^3^ cells of each clone were suspended in culture medium containing 10% FBS and 0.35% Bacto agar (Amresco, OH, USA). The agar cell mixture was plated on top of a bottom layer with 0.6% agar‐medium mixture in six‐well plates. After 14–21 days, cell colonies were fixed and stained with 0.005% Crystal Violet for 1 h. The photographs of the cells growing in the plate and the colonies developed in soft agar were taken, the number of colonies larger than 0.5 mm was scored by ImageJ V1.45 (NIH, USA).

### Colony Formation Assay

H1299, A549 and H1975 were trypsinized into single‐cell status and plated in 6‐well plates at a density of 300 cells per well. After 14 days, cell colonies were fixed with methanol and stained with 0.5% Crystal Violet. The photographs of colonies were taken, and the number of colonies larger than 200 µm was scored by ImageJ V1.8.0 (NIH, USA). Each experiment was performed in triplicate.

### 3D Culture Growth Assays

The 3D culture growth assays was performed as previously described.^[^ ^77^ ^]^ Briefly, 5 µl of cells (1 × 10^5^ cells ml^−1^) mixed with 5 µl of 3D culture matrix (#3445‐005‐01, Trevigen, Gaithersburg, MD, USA) were added into the inner well of µ‐slides (ibidi Gmbh, Martinsried, Germany) and incubated for at least 60 min at 37 °C. After polymerization, cell‐free medium were added to fill the upper well. Microscopy images were taken after three days or longer time.

### Vasculogenic Mimicry (VM) Formation

For vasculogenic mimicry assay, matrigel matrix pre‐thawed at 4 °C were added into the inner well of µ‐slides and incubated for at least 30 min at 37 °C until polymerization. 50 µl of cells (1 × 10^5^ cells ml^−1^) were added onto the polymerizd matrix. Microscopy images were taken after 24 h.

### In Vitro Kinase Reaction Assay

Purified TBK1 protein were prepared as following. Briefly, cells expressing Flag‐TBK1 were lysed in cell lysis buffer [20 mM Tris‐HCl (pH 7.5), 150 mM NaCl, 1 mM EDTA, 1 mM EGTA, 1% Triton, 2.5 mM sodium pyrophosphate, 1 mM beta‐glycerophosphate, 1 mM Na_3_VO_4_, 1 mM PMSF and Complete Protease Inhibitor Cocktail) for 1 h on ice. Lysates were centrifuged for 30 min at 4 °C after sonication, and then, the supernatants were transferred into new tubes and incubated with anti‐Flag M2 affinity beads overnight at 4 °C. The beads were washed three times and Flag‐TBK1 were purified using the 3×Flag peptide according to the manufacturer's specifications (Sigma).

Kinase reactions were performed by incubation of Flag‐TBK1 in the kinase buffer [25 mM Tris‐HCl (pH 7.5), 5 mM beta‐glycerophosphate, 2 mM dithiothreitol (DTT), 0.1 mM Na_3_VO_4_, 10 mM MgCl_2_] with 1 mM ATP and 1.0 µg purified GST‐fused AGO2 protein as the substrate at 30 °C for 90 min in 50‐µl reaction mixture. Samples were separated by SDS‐PAGE, and analyzed by immunoblotting with AGO2 specific phospho‐antibody (pS417‐AGO2) or anti‐pan‐specific phospho‐Ser/Thr antibody.

### Animal Experiments

For xenografted tumor models, each of H1299 stable cell lines in 100 µL Opti‐MEM medium containing 2.5 × 10^6^ cells were injected subcutaneously into 6‐week‐old BALB/c nude mice individually. Mice were killed after 4 weeks and tumors were weighted and photographed.

To investigate the therapeutic role of oncogenic miRNAs, H1299 cells in 100 µL Opti‐MEM medium containing 2.5 × 10^6^ cells were subcutaneously injected into nude mice. After 7 days, mice were randomly divided into four groups (5 mice/group). Once tumors reached an average of 5 mm × 5 mm, the mice were treated with 5 nmol antagomiR‐NC, antagomiR‐21 or antagomiRs‐21+9+10b by intratumoral injection and TBK1 inhibitor Amlexanox (25 mg k^−1^ g) by intraperitoneal injection for twice per week. Mice were sacrificed on day 14 after treatment, tumors were photographed, size measured and weighted.

To explore the potential of combination therapy as a strategy to drug‐resistant lung cancer cells in vivo, PC9/GR cells in 100 µL Opti‐MEM medium containing 4 × 10^6^ cells were subcutaneously injected into nude mice. Mice were randomly divided into four groups (5 mice/group). Once tumors reached an average of 5 mm × 5 mm, the mice were treated with Gefitinb (20 mg k^−1^g) by oral gavage, Amlexanox (20 mg k^−1^ g) by intraperitoneal injection, and combination therapy for every 2 days. Luciferase‐expressing H1975^luc^ cells in 100 µL Opti‐MEM medium containing 2.5 × 10^6^ cells were subcutaneously injected into nude mice. After 7 days, mice were randomly divided into four groups (5 mice/group). Once tumors reached an average of 5 mm × 5 mm, the mice were treated with Gefitinb (20 mg k^−1^ g) by oral gavage, Amlexanox (25 mg k^−1^ g) by intraperitoneal injection, and combination therapy for every 3 days. The luciferase signal intensity was monitored in vivo by bio‐luminescence imaging (BLI) before and after treatment. Mice were sacrificed on day 14 after treatment, tumors were photographed, size measured and weighted.

All animal studies were conducted with the approval and guidance of Shanghai Jiao Tong University Medical Animal Ethics Committees, and the assigned approval number for the ethical clearance to conduct animal experiments is #A‐2022‐067.

### Mass Spectrometry

To identify phosphorylation sites of AGO2, Flag‐AGO2 precipitated from 293T cells co‐transfected with TBK1 was analyzed by Coomassie Blue staining. The protein band corresponding to AGO2 was excised and subjected to in‐gel digestion with tryspin. Samples were analyzed by Ultimate Capillary LC system (Dionex) directly coupled to LTQ Orbitrap Mass Analyzer using the TopTenTM method. The data was searched on MASCOT (MassMatrix) against the human Swiss‐Prot database. All the identified phospho‐peptides were further confirmed by manually checking the results.

### Phos‐Tag SDS–PAGE Electrophoresis

SDS–PAGE gels (8%) were supplemented with 50 µM Phos‐tag AAL solution (Wako) according to the manufacturer's recommendations. Gels were run at 100 V until the dye front completely exited the gel. Gels were incubated in transfer buffer supplemented with 10 mM EDTA for 10 min. Gels were then soaked in normal transfer buffer for 10 min. Proteins were transferred to a PVDF membrane and standard Western blotting procedures were subsequently followed.

### RIP‐Seq/miRNA‐Seq/RNA‐Seq

RIP‐Seq/miRNA‐Seq and subsequent bioinformatics analysis were all done by Cloud‐Seq Biotech (Shanghai, China). Cells were lysed in an ice‐cold lysis buffer. Then, RNA immunoprecipitation (RIP) was performed with the GenSeqTM RIP Kit (GenSeq, China). miRNAs bound to AGO2, and total miRNAs (as an input) were extracted using Trizol by following manufacturer's instruction (Thermo Fisher Scientific), and then used for the preparation of the miRNA sequencing library, which including 3′‐adaptor ligation, 5′‐adaptor ligation, cDNA synthesis and PCR amplifification, ≈150 bp PCR amplicons (corresponding to ≈22 nt miRNAs) size of products were selected and purified. miRNA‐Seq libraries sequencing were denatured as single‐stranded DNA molecules, captured on Illumina flow cells, amplified in situ as clusters and finally sequenced for 50 cycles on Illumina HiSeq Sequencer according to the manufacturer's instructions.

For RNA‐Seq, total RNAs were extracted by using TRIZOL reagent as following manufacturer's instruction (Invitrogen), then the rRNAs were removed from the immunoprecipitated RNA and input RNA samples by using RNAs with NEBNext rRNA Depletion Kit (New England Biolabs, Inc., Massachusetts, USA). The rRNA‐depleted RNAs were constructed RNA sequencing libraries by using NEBNext Ultra II Directional RNA Library Prep Kit (New England Biolabs, Inc.,

Massachusetts, USA) according to the manufacturer's instructions. RNA‐Seq libraries were controlled for quality and quantified using the BioAnalyzer 2100 system (Agilent Technologies, Inc., USA), and the libraries sequencing were performed on an Illumina Hiseq instrument with 150 bp paired‐end reads.

### Analyses for High‐Throughput Sequencing Data

For RIP‐Seq/miRNA‐Seq, raw data were generated after sequencing, image analysis, base calling and quality filtering on Illumina sequencer and finally quality controlled by Q30. The adaptor sequences were trimmed and the adaptor‐trimmed‐reads (> = 15 nt) were left by cut adapt software (version 1.9.2). Then the trimmed reads from all samples were pooled, and miRDeep2 software (version 2.0.0.5) was used to predict novel miRNAs. The trimmed reads were aligned to the merged human miRNA databases using Novoalign software (version 3.02.12) with at most one mismatch. The numbers of mature miRNA mapped tags were defined as the raw expression levels of that miRNA. The read counts were normalized by TPM (tag counts per million aligned miRNAs) approach. Differentially expressed miRNAs between two samples were filtered through Fold change.

For RNA‐Seq, paired‐end reads were harvested from Illumina HiSeq 4000 sequencer, and were quality controlled by Q30. After 3′ adaptor‐trimming and low quality reads removing by cutadapt software (v1.9.3). The high quality reads were aligned to the human reference genome (UCSC hg19) with hisat2 software (v2.0.4). Then, guided by the Ensembl gtf gene annotation file, cuffdiff software (v2.2.1, part of cufflinks) was used to get the FPKM as the expression profiles of mRNA, and fold change were calculated based on FPKM, differentially expressed mRNA were identified.

### Clinical Lung Cancer Specimens

Clinical lung cancer tissue arrays were purchased from bioaitech (Catalog # R121Lu01) and ALenabio (Catalog #DC‐Lun01032 and DC‐Lun01053, Xi'an, China). The utilization of clinical NSCLC specimens from Ren Ji Hospital (School of Medicine, Shanghai Jiao Tong University, Shanghai, China) for the study was approved by the ethics committee of Ren Ji Hospital. The testing of clinical samples strictly followed the protocol approved by the Renji Hospital Ethics Committees. The assigned approval number of the ethical approval is #RA‐2022‐159.

### Immunohistochemical Staining

IHC staining was performed by using antibodies against pS417‐AGO2 (1:50) and AGO2 (1:50, abcam,#ab57113). Staining index score was determined by multiplying the intensity score (no staining: 0; weak staining: 1, moderate staining: 2 and strong staining: 3) and the score for the extent (0%–5%: 0; 5%–25%: 1; 26%–50%: 2; 51%–75%: 3 and 76%–100%: 4) of stained cells, generating a score that ranged from 0 (the minimum score) to 12 (the maximum score). Histochemistry score (H score) was calculated basing on the percentage and intensity of positivelystained cells, following equation: H‐Score = ∑(pi × i) = (percentage of weak intensity cells × 1) + (percentage of moderate intensity cells × 2) + (per centage of strong intensity cells × 3), where i is the intensity of the stained cells (0 to 3), and Pi is the percentage of stained cells.

### Statistical Analysis

All statistical analyses were performed with the GraphPad Prism 8.0 software package. Data in this work were presented as the mean ± standard deviation (n ≥ 3). Statistical evaluations between two groups were performed by Student's t‐test. Experiments with more than three groups were evaluated by one‐way ANOVA. The cumulative fraction analysis was performed by a two sided Mann–Whitney U test. The survival curve was established by the Kaplan–Meier method and compared by the log‐rank test. *p* < 0.05(*), *p* < 0.01(**), *p* < 0.001(***) and *p* < 0.0001(****) were considered statistically significant in all cases.

## Conflict of Interest

The authors declare no conflict of interest.

## Author Contributions

J.Y., X.Z. and Y.F. designed the study; Y.C., R.L., Z.Z., C.H., L.L., J.H, R.C., Y.W., and J.H. performed experiments; J.C. and J.Z. analyzed data; J.Y. and X.Z. wrote the manuscript; J.Y. oversaw the project.

## Supporting information

Supporting Information

## Data Availability

The data that support the findings of this study are available on request from the corresponding author. The data are not publicly available due to privacy or ethical restrictions.

## References

[1] R. L. Siegel , K. D. Miller , N. S. Wagle , A. Jemal , CA Cancer J Clin 2023, 73, 17. 36633525 10.3322/caac.21763

[2] L. Gandhi , M. C. Garassino , N. Engl. J. Med. 2018, 379, e18. 10.1056/NEJMc1808567 30207917

[3] X. Sun , S. Xu , Z. Yang , P. Zheng , W. Zhu , Expert Opin. Ther. Pat. 2021, 31, 223. 33315482 10.1080/13543776.2021.1860210

[4] H. Yang , S. Q. Liang , R. A. Schmid , R. W. Peng , Front Oncol 2019, 9, 953. 31612108 10.3389/fonc.2019.00953 PMC6773824

[5] M. G. Denis , J. Bennouna , Cancer Manag Res 2020, 12, 12593. 33324104 10.2147/CMAR.S218751 PMC7733376

[6] H. Burnett , H. Emich , C. Carroll , N. Stapleton , P. Mahadevia , T. Li , PLoS One 2021, 16, e0247620. 33684140 10.1371/journal.pone.0247620 PMC7939356

[7] J. K. Sabari , B. H. Lok , J. H. Laird , J. T. Poirier , C. M. Rudin , Nat. Rev. Clin. Oncol. 2017, 14, 549. 28534531 10.1038/nrclinonc.2017.71 PMC5843484

[8] P. Khan , J. A. Siddiqui , S. K. Maurya , I. Lakshmanan , M. Jain , A. K. Ganti , R. Salgia , S. K. Batra , M. W. Nasser , Semin. Cancer Biol. 2020, 57, 10.1016/j.semcancer.2020.11.006. PMC8218609 33220460

[9] N. Vasan , J. Baselga , D. M. Hyman , Nature 2019, 575, 299. 31723286 10.1038/s41586-019-1730-1 PMC8008476

[10] K. Shah , R. M. Rawal , Curr. Drug Metab. 2019, 20, 1114. 31902353 10.2174/1389200221666200103111539

[11] M. Chen , L. Wang , F. Wang , F. Li , W. Xia , H. Gu , Y. Chen , Int J Nanomedicine 2019, 14, 3557. 31190812 10.2147/IJN.S198511 PMC6526930

[12] M. Das , S. Musetti , L. Huang , Nucleic Acid Ther. 2019, 29, 61. 30562145 10.1089/nat.2018.0762 PMC6461149

[13] F. Pastor , P. Berraondo , I. Etxeberria , J. Frederick , U. Sahin , E. Gilboa , I. Melero , Nat Rev Drug Discov 2018, 17, 751. 30190565 10.1038/nrd.2018.132

[14] R. L. Juliano , NAR Cancer 2020, 2, zcaa025. 33015625 10.1093/narcan/zcaa025 PMC7520847

[15] X. Zhang , K. Xie , H. Zhou , Y. Wu , C. Li , Y. Liu , Z. Liu , Q. Xu , S. Liu , D. Xiao , Y. Tao , Mol Cancer 2020, 19, 47. 32122355 10.1186/s12943-020-01171-z PMC7050132

[16] D. P. Bartel , Cell 2018, 173, 20.29570994

[17] B. Ortiz‐Quintero , Cancers (Basel) 2020, 12. 10.3390/cancers12113455 PMC7699762 33233600

[18] A. A. Svoronos , D. M. Engelman , F. J. Slack , Cancer Res. 2016, 76, 3666. 27325641 10.1158/0008-5472.CAN-16-0359 PMC4930690

[19] M. J. Bueno , M. Malumbres , Biochim. Biophys. Acta 2011, 1812, 592. 21315819 10.1016/j.bbadis.2011.02.002

[20] G. S. Heyn , L. H. Correa , K. G. Magalhaes , Front Endocrinol (Lausanne) 2020, 11, 563816. 33123088 10.3389/fendo.2020.563816 PMC7573351

[21] J. Eniafe , S. Jiang , Wiley Interdiscip Rev RNA 2021, 12, e1635. 33230974 10.1002/wrna.1635

[22] H. Li , J. Zhao , X. Jia , Y. Zhang , Y. Du , H. Li , L. Ma , J. Huang , Int J Clin Exp Pathol 2020, 13, 692. 32355517 PMC7191137

[23] J. Liao , J. Shen , Q. Leng , M. Qin , M. Zhan , F. Jiang , Thorac Cancer 2020, 11, 762. 31994346 10.1111/1759-7714.13337 PMC7049510

[24] Z. Shen , X. Xu , L. Lv , H. Dai , J. Chen , B. Chen , Gastroenterol Res Pract 2020, 2020, 6478653. 33193757 10.1155/2020/6478653 PMC7641708

[25] G. Wang , Y. Zhou , W. Chen , Y. Yang , J. Ye , H. Ou , H. Wu , Cancer Biomark. 2020, 28, 549. 32623387 10.3233/CBM-201489 PMC12662378

[26] Y. N. Liu , M. F. Tsai , S. G. Wu , T. H. Chang , T. H. Tsai , C. H. Gow , H. Y. Wang , J. Y. Shih , Mol Ther Nucleic Acids 2020, 22, 471. 33230450 10.1016/j.omtn.2020.09.015 PMC7554328

[27] I. Haque , H. I. Kawsar , H. Motes , M. Sharma , S. Banerjee , S. K. Banerjee , A. K. Godwin , C. H. Huang , Int. J. Mol. Sci. 2020, 21. 10.3390/ijms21239307 PMC7729622 33291316

[28] V. N. Kim , J. Han , M. C. Siomi , Nat. Rev. Mol. Cell Biol. 2009, 10, 126. 19165215 10.1038/nrm2632

[29] P. B. Kwak , Y. Tomari , Nat. Struct. Mol. Biol. 2012, 19, 145. 22233755 10.1038/nsmb.2232

[30] R. I. Gregory , T. P. Chendrimada , N. Cooch , R. Shiekhattar , Cell 2005, 123, 631. 16271387 10.1016/j.cell.2005.10.022

[31] I. J. MacRae , E. Ma , M. Zhou , C. V. Robinson , J. A. Doudna , Proc Natl Acad Sci U S A 2008, 105, 512. 18178619 10.1073/pnas.0710869105 PMC2206567

[32] C. Chen , C. Zhu , J. Huang , X. Zhao , R. Deng , H. Zhang , J. Dou , Q. Chen , M. Xu , H. Yuan , Y. Wang , J. Yu , Nat. Commun. 2015, 6, 8899. 26582366 10.1038/ncomms9899 PMC4673853

[33] T. Kawamata , H. Seitz , Y. Tomari , Nat. Struct. Mol. Biol. 2009, 16, 953. 19684602 10.1038/nsmb.1630

[34] J. H. Park , C. Shin , Nucleic Acids Res. 2015, 43, 9418. 26384428 10.1093/nar/gkv937 PMC4627090

[35] M. Yoda , T. Kawamata , Z. Paroo , X. Ye , S. Iwasaki , Q. Liu , Y. Tomari , Nat. Struct. Mol. Biol. 2010, 17, 17. 19966796 10.1038/nsmb.1733 PMC2915567

[36] J. C. Medley , G. Panzade , A. Y. Zinovyeva , Wiley Interdiscip Rev RNA 2021, 12, e1627. 32954644 10.1002/wrna.1627 PMC8047885

[37] H. Zhang , X. Zhao , Y. Guo , R. Chen , J. He , L. Li , Z. Qiang , Q. Yang , X. Liu , C. Huang , R. Lu , J. Fang , Y. Cao , J. Huang , Y. Wang , J. Huang , G. Q. Chen , J. Cheng , J. Yu , Nat. Commun. 2021, 12, 5416. 34518544 10.1038/s41467-021-25739-5 PMC8438024

[38] J. Hiscott , Cytokine Growth Factor Rev. 2007, 18, 483. 17706453 10.1016/j.cytogfr.2007.06.002

[39] X. Ma , E. Helgason , Q. T. Phung , C. L. Quan , R. S. Iyer , M. W. Lee , K. K. Bowman , M. A. Starovasnik , E. C. Dueber , Proc Natl Acad Sci U S A 2012, 109, 9378. 22619329 10.1073/pnas.1121552109 PMC3386122

[40] L. Zhu , Y. Li , X. Xie , X. Zhou , M. Gu , Z. Jie , C. J. Ko , T. Gao , B. E. Hernandez , X. Cheng , S. C. Sun , Nat. Cell Biol. 2019, 21, 1604. 31792381 10.1038/s41556-019-0429-8 PMC6901116

[41] C. Shu , B. Sankaran , C. T. Chaton , A. B. Herr , A. Mishra , J. M. Peng , P. W. Li , Structure 2013, 21, 1137. 23746807 10.1016/j.str.2013.04.025 PMC3702631

[42] J. G. Zhang , J. J. Wang , F. Zhao , Q. Liu , K. Jiang , G. H. Yang , Clin. Chim. Acta 2010, 411, 846. 20223231 10.1016/j.cca.2010.02.074

[43] Y. S. Lee , A. Dutta , Genes Dev. 2007, 21, 1025. 17437991 10.1101/gad.1540407 PMC1855228

[44] K. Miyoshi , H. Uejima , T. Nagami‐Okada , H. Siomi , M. C. Siomi , Methods Mol Biol 2008, 442, 29. 18369776 10.1007/978-1-59745-191-8_3

[45] K. Miyoshi , T. N. Okada , H. Siomi , M. C. Siomi , RNA 2009, 15, 1282. 19451544 10.1261/rna.1541209 PMC2704077

[46] J. L. Johnson , T. M. Yaron , E. M. Huntsman , A. Kerelsky , J. Song , A. Regev , T. Y. Lin , K. Liberatore , D. M. Cizin , B. M. Cohen , N. Vasan , Y. Ma , K. Krismer , J. T. Robles , B. van de Kooij , A. E. van Vlimmeren , N. Andree‐Busch , N. F. Kaufer , M. V. Dorovkov , A. G. Ryazanov , Y. Takagi , E. R. Kastenhuber , M. D. Goncalves , B. D. Hopkins , O. Elemento , D. J. Taatjes , A. Maucuer , A. Yamashita , A. Degterev , M. Uduman , et al., Nature 2023, 613, 759. 36631611 10.1038/s41586-022-05575-3 PMC9876800

[47] C. Mo , H. Ha , X. Tang , X. Lu , Y. Wei , D. Luo , Z. Zhou , Panminerva Med. 2020, 10.23736/S0031-0808.20.03937-3. 32495612

[48] A. Khandwala , R. G. Van Inwegen , M. R. Charney , M. C. Alfano , Oral Surg Oral Med Oral Pathol Oral Radiol Endod 1997, 83, 231. 9117755 10.1016/s1079-2104(97)90010-x

[49] B. Li , S. X. Ren , X. F. Li , Y. S. Wang , D. Garfield , S. W. Zhou , X. X. Chen , C. X. Su , M. Chen , P. Kuang , G. H. Gao , Y. Y. He , L. H. Fan , K. Fei , C. C. Zhou , G. Schmit‐Bindert , Lung Cancer 2014, 83, 146. 24331411 10.1016/j.lungcan.2013.11.003

[50] J. Y. Hu , S. L. Huang , X. L. Liu , Y. Zhang , S. L. Wei , X. H. Hu , J Immunol Res 2022, 2022.

[51] E. Tili , J. J. Michaille , D. Wernicke , H. Alder , S. Costinean , S. Volinia , C. M. Croce , P Natl Acad Sci USA 2011, 108, 4908. 10.1073/pnas.1101795108 PMC3064319 21383199

[52] W. W. Du , W. Yang , L. Fang , J. Xuan , H. Li , A. Khorshidi , S. Gupta , X. Li , B. B. Yang , Cell Death Dis. 2014, 5. 10.1038/cddis.2014.305 PMC4123096 25077541

[53] M. Garofalo , G. Romano , G. Di Leva , G. Nuovo , Y. J. Jeon , A. Ngankeu , J. Sun , F. Lovat , H. Alder , G. Condorelli , J. A. Engelman , M. Ono , J. K. Rho , L. Cascione , S. Volinia , K. P. Nephew , C. M. Croce , Nat. Med. 2014, 20, 103.

[54] J. Krutzfeldt , N. Rajewsky , R. Braich , K. G. Rajeev , T. Tuschl , M. Manoharan , M. Stoffel , Nature 2005, 438, 685. 16258535 10.1038/nature04303

[55] S. L. Monica , D. Madeddu , M. Tiseo , V. Vivo , M. Galetti , D. Cretella , M. Bonelli , C. Fumarola , A. Cavazzoni , A. Falco , A. Gervasi , C. A. Lagrasta , N. Naldi , E. Barocelli , A. Ardizzoni , F. Quaini , P. G. Petronini , R. Alfieri , J Thorac Oncol 2016, 11, 1051. 27006151 10.1016/j.jtho.2016.03.006

[56] Y. Hu , J. Zang , X. Qin , D. Yan , H. Cao , L. Zhou , J. Ni , S. Yu , J. Wu , J. F. Feng , Onco Targets Ther 2017, 10, 2341. 28496332 10.2147/OTT.S124757 PMC5417672

[57] A. Rybak , H. Fuchs , K. Hadian , L. Smirnova , E. A. Wulczyn , G. Michel , R. Nitsch , D. Krappmann , F. G. Wulczyn , Nat. Cell Biol. 2009, 11, 1411. 19898466 10.1038/ncb1987

[58] S. Zhang , X. Zhang , Y. Bie , J. Kong , A. Wang , Y. Qiu , X. Zhou , Virol Sin 2022, 37, 569. 35533808 10.1016/j.virs.2022.05.001 PMC9437610

[59] J. Han , C. A. LaVigne , B. T. Jones , H. Zhang , F. Gillett , J. T. Mendell , Science 2020, 370. 33184234 10.1126/science.abc9546 PMC8177725

[60] C. Y. Shi , E. R. Kingston , B. Kleaveland , D. H. Lin , M. W. Stubna , D. P. Bartel , Science 2020, 370. 33184237 10.1126/science.abc9359 PMC8356967

[61] H. H. Qi , P. P. Ongusaha , J. Myllyharju , D. Cheng , O. Pakkanen , Y. Shi , S. W. Lee , J. Peng , Y. Shi , Nature 2008, 455, 421. 18690212 10.1038/nature07186 PMC2661850

[62] A. K. Leung , S. Vyas , J. E. Rood , A. Bhutkar , P. A. Sharp , P. Chang , Mol. Cell 2011, 42, 489. 21596313 10.1016/j.molcel.2011.04.015 PMC3898460

[63] H. Zhang , Y. Wang , J. Dou , Y. Guo , J. He , L. Li , X. Liu , R. Chen , R. Deng , J. Huang , R. Xie , X. Zhao , J. Yu , Oncogene 2019, 38, 1410. 30305728 10.1038/s41388-018-0530-7 PMC6372475

[64] S. Rudel , Y. Wang , R. Lenobel , R. Korner , H. H. Hsiao , H. Urlaub , D. Patel , G. Meister , Nucleic Acids Res. 2011, 39, 2330. 21071408 10.1093/nar/gkq1032 PMC3064767

[65] J. Shen , W. Xia , Y. B. Khotskaya , L. Huo , K. Nakanishi , S. O. Lim , Y. Du , Y. Wang , W. C. Chang , C. H. Chen , J. L. Hsu , Y. Wu , Y. C. Lam , B. P. James , X. Liu , C. G. Liu , D. J. Patel , M. C. Hung , Nature 2013, 497, 383. 23636329 10.1038/nature12080 PMC3717558

[66] T. Liu , H. Zhang , J. Fang , Z. Yang , R. Chen , Y. Wang , X. Zhao , S. Ge , J. Yu , J. Huang , Neoplasia 2020, 22, 129. 31981897 10.1016/j.neo.2019.12.004 PMC6992904

[67] S. R. Horman , M. M. Janas , C. Litterst , B. Wang , I. J. MacRae , M. J. Sever , D. V. Morrissey , P. Graves , B. Luo , S. Umesalma , H. H. Qi , L. J. Miraglia , C. D. Novina , A. P. Orth , Mol. Cell 2013, 50, 356. 23603119 10.1016/j.molcel.2013.03.015 PMC3654076

[68] R. J. Golden , B. Chen , T. Li , J. Braun , H. Manjunath , X. Chen , J. Wu , V. Schmid , T. C. Chang , F. Kopp , A. Ramirez‐Martinez , V. S. Tagliabracci , Z. J. Chen , Y. Xie , J. T. Mendell , Nature 2017, 542, 197. 28114302 10.1038/nature21025 PMC5302127

[69] M. Quevillon Huberdeau , D. M. Zeitler , J. Hauptmann , A. Bruckmann , L. Fressigne , J. Danner , S. Piquet , N. Strieder , J. C. Engelmann , G. Jannot , R. Deutzmann , M. J. Simard , G. Meister , EMBO J. 2017, 36, 2088. 28645918 10.15252/embj.201696386 PMC5510005

[70] A. P. Runde , R. Mack , J. P. S. , J. Zhang , J Exp Clin Cancer Res 2022, 41, 135. 35395857 10.1186/s13046-022-02352-y PMC8994244

[71] J. M. Cooper , Y. H. Ou , E. A. McMillan , R. M. Vaden , A. Zaman , B. O. Bodemann , G. Makkar , B. A. Posner , M. A. White , Cancer Res. 2017, 77, 5077. 28716898 10.1158/0008-5472.CAN-17-0829 PMC5833933

[72] J. D. Campbell , A. Alexandrov , J. Kim , J. Wala , A. H. Berger , C. S. Pedamallu , S. A. Shukla , G. Guo , A. N. Brooks , B. A. Murray , M. Imielinski , X. Hu , S. Ling , R. Akbani , M. Rosenberg , C. Cibulskis , A. Ramachandran , E. A. Collisson , D. J. Kwiatkowski , M. S. Lawrence , J. N. Weinstein , R. G. Verhaak , C. J. Wu , P. S. Hammerman , A. D. Cherniack , G. Getz , N. Cancer Genome Atlas Research , M. N. Artyomov , R. Schreiber , R. Govindan , et al., Nat. Genet. 2016, 48, 607. 27158780 10.1038/ng.3564 PMC4884143

[73] D. Bautista‐Sanchez , C. Arriaga‐Canon , A. Pedroza‐Torres , I. A. De La Rosa‐Velazquez , R. Gonzalez‐Barrios , L. Contreras‐Espinosa , R. Montiel‐Manriquez , C. Castro‐Hernandez , V. Fragoso‐Ontiveros , R. M. Alvarez‐Gomez , L. A. Herrera , Mol Ther Nucleic Acids 2020, 20, 409. 32244168 10.1016/j.omtn.2020.03.003 PMC7118281

[74] X. J. Gu , Y. Y. Qiu , M. Lin , K. Cui , G. X. Chen , Y. Z. Chen , C. C. Fan , Y. M. Zhang , L. Xu , H. Z. Chen , J. B. Wan , W. Lu , Z. Y. Xiao , Nano Lett. 2019, 19, 3344. 30974946 10.1021/acs.nanolett.9b01065

[75] J. Dou , H. Zhang , R. Chen , Z. Shu , H. Yuan , X. Zhao , Y. Wang , J. Huang , A. Zhou , J. Yu , Mol. Oncol. 2020, 14, 2288. 32333719 10.1002/1878-0261.12694 PMC7463354

[76] Y. Du , G. Hou , H. Zhang , J. Dou , J. He , Y. Guo , L. Li , R. Chen , Y. Wang , R. Deng , J. Huang , B. Jiang , M. Xu , J. Cheng , G. Q. Chen , X. Zhao , J. Yu , Nucleic Acids Res. 2018, 46, 5195. 29506078 10.1093/nar/gky156 PMC6007514

[77] G. Hou , X. Zhao , L. Li , Q. Yang , X. Liu , C. Huang , R. Lu , R. Chen , Y. Wang , B. Jiang , J. Yu , Nucleic Acids Res. 2021, 49, 2859. 33577677 10.1093/nar/gkab065 PMC7969013

