# Supplementary material for: Phosphorylation of AGO2 by TBK1 Promotes the Formation of Oncogenic miRISC in NSCLC
Source: Adv Sci (Weinh). 2024 Feb 13;11(15):2305541. doi: 10.1002/advs.202305541 (PMC11022703; doi:10.1002/advs.202305541)
Supplement: Supplementary file 1 — Supporting Information [file ADVS-11-2305541-s001.pdf]

ADVANCED SCIENCE
Open Access

Supporting Information

for *Adv. Sci.*, DOI 10.1002/advs.202305541

Phosphorylation of AGO2 by TBK1 Promotes the Formation of Oncogenic miRISC in NSCLC

Xian Zhao, Yingting Cao, Runhui Lu, Zihan Zhou, Caihu Huang, Lian Li, Jiayi Huang, Ran Chen, Yanli Wang, Jian Huang, Jinke Cheng, Junke Zheng, Yujie Fu* and Jianxiu Yu**

Zhao_Fig.S1

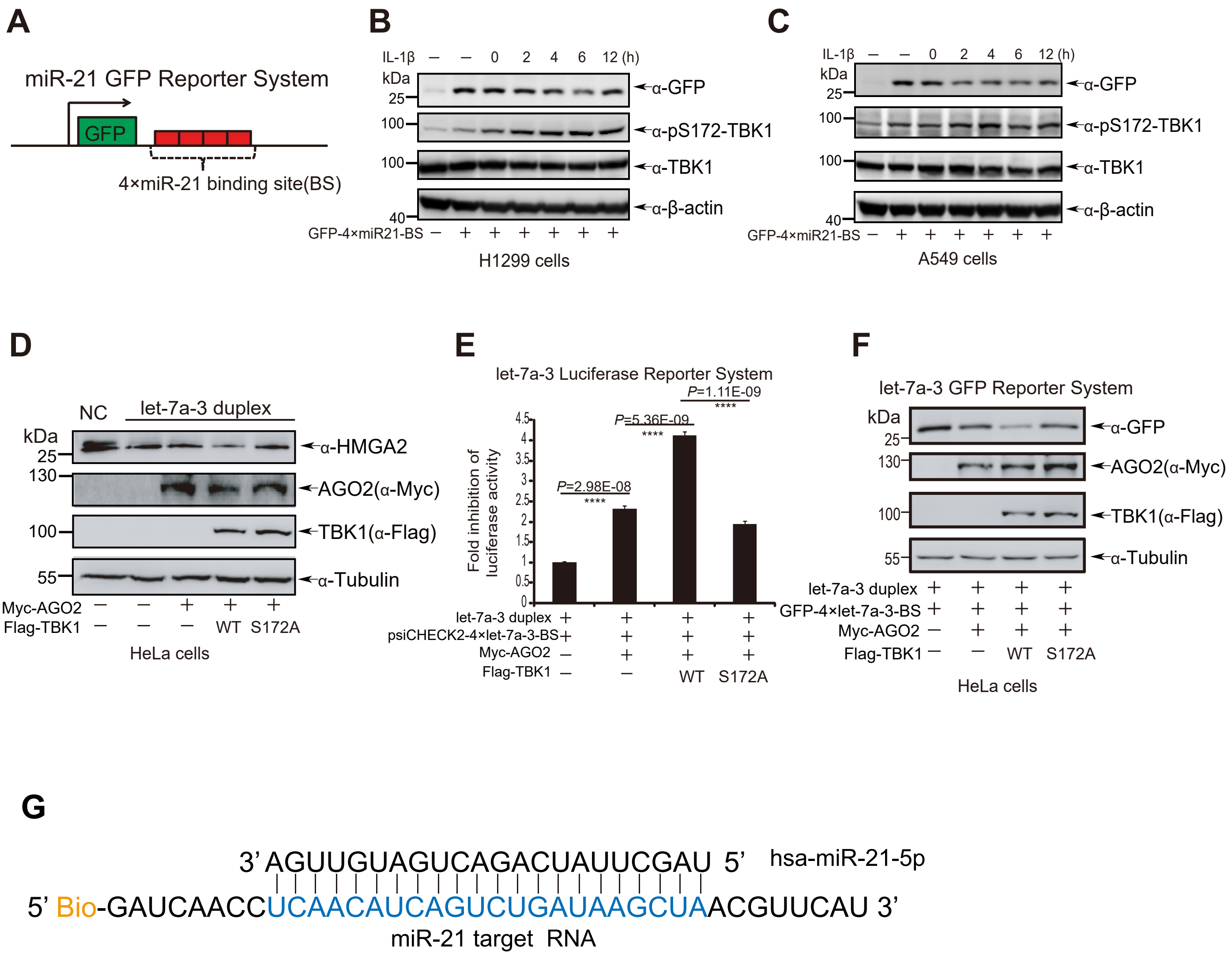

Figure S1. TBK1 promotes miRNA loading and miRNA-guided gene silence, Related to Figure 1.
(A) A schematic illustration of the miR-21 GFP Reporter System. (B-C) H1299 (B) or A549 (C) cells transfected with GFP-4×miR-21-BS were treated with IL-1β (10 ng/ml) for the indicated time, and then harvested for Western blotting analysis with the indicated antibodies (Related to Fig.1A). (D) Lysates form HeLa cells co-transfected let-7a-3 mimics, Myc-AGO2 with Flag-TBK1^{WT}or TBK1^{S172A} were used for western blotting analysis with anti-HMGA2 antibody. (E) 293T cells co-transfected let-7a-3 dup**lex**, psiCHECK2-4×let-7a-BS, Myc-AGO2 with Flag-TBK1^{WT} or TBK1^{S172A} were harvested for the dual-luciferase activity assay. The Renilla luciferase values were normalized to the Firefly luciferase activity and plotted as relative luciferase activity. Data were presented as mean ± SD, n = 3 biologically independent samples. Statistical analysis was performed using one-way ANOVA. ****$p < 0.0001$. (F) HeLa cells co-transfected let-7a-3 duplex, GFP-4×let-7a-3-BS, Myc-AGO2 with Flag-TBK1^{WT} or TBK1^{S172A} were harvested for Western blotting analysis with anti-GFP antibody. (G) Schematic showing the sequence information of the Bio-miR-21 target RNA.

Zhao_Fig.S2

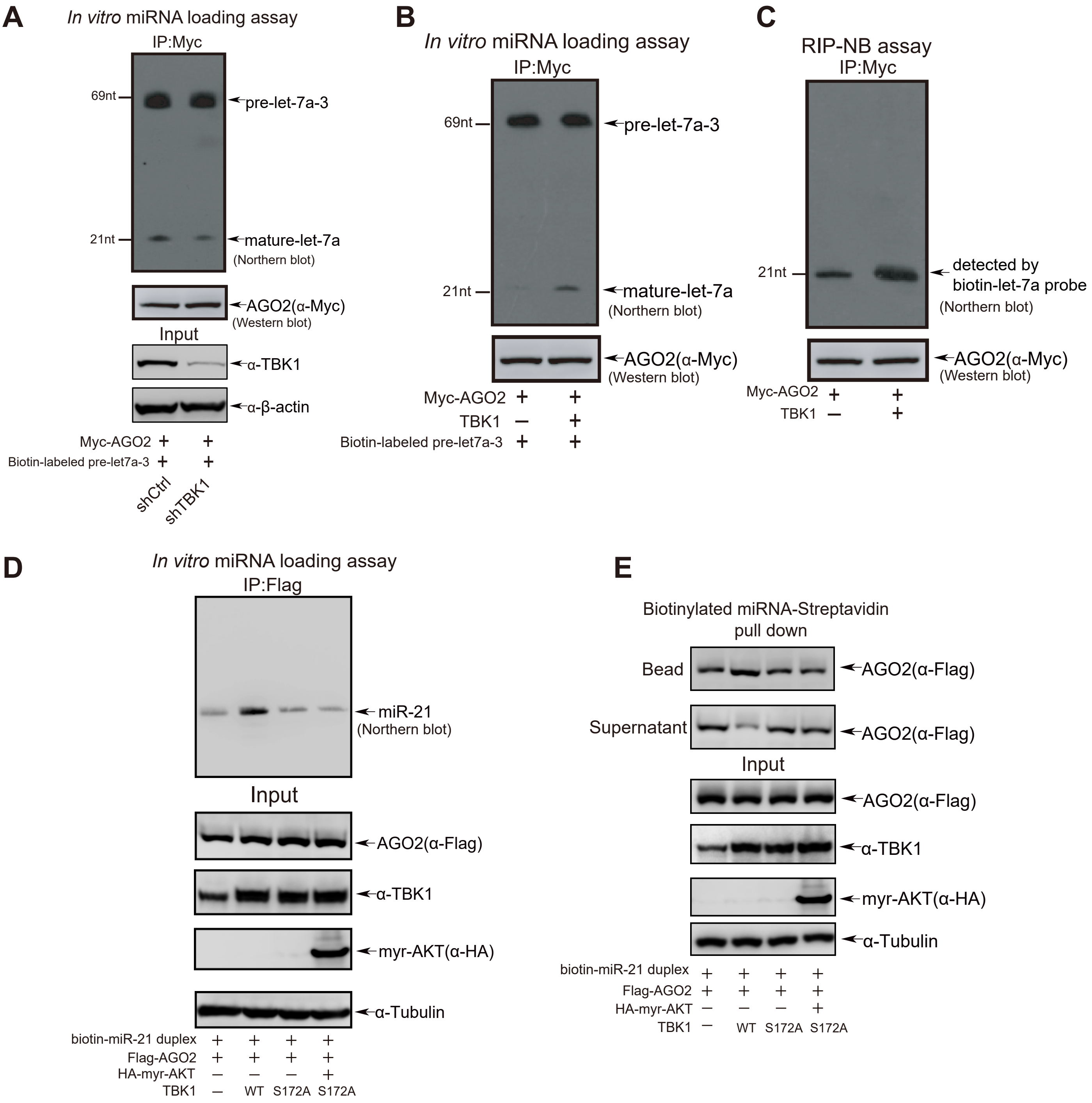

Figure S2. TBK1 promotes miRNA loading, Related to Figure 2.
(A) Lysates from 293T-shCtrl and 293T-shTBK1 cells transfected with Myc-AGO2 were immunoprecipitated by Myc-AGO2, respectively, and then the beads coupled with AGO2 were co-incubated with biotin-tagged pre-let-7a for in vitro miRNA loading assay. The let-7a associated with AGO2 was detected by Northern blotting analysis with biotin-let-7a probe. (B) Lysates from 293T cells co-transfected Myc-AGO2 with or without TBK1 were immunoprecipitated by Myc-AGO2, then the beads were co-incubated with biotin-tagged pre-let-7a for in vitro miRNA loading assay. The let-7a associated with AGO2 was detected by Northern blotting analysis with biotin-let-7a probe. (C) 293T cells co-transfected Myc-AGO2 with or without TBK1 were lysed for the RIP assay with anti-Myc antibody, and then let-7a associated with AGO2 was detected by Northern blotting analysis with biotin-let-7a probe. (D-E) 293T cells were co-transfected with Flag-AGO2 along with $TBK1^{WT}$, $TBK1^{S172A}$, or $TBK1^{S172A}$ and myr-AKT (a constitutively active form of AKT featuring an N-terminally attached myristoylation signal). Lysates were immunoprecipitated by Flag-AGO2, then the beads were co-incubated with purified and biotin-tagged miR-21 duplex for *in vitro* miRNA loading assay (D). Lysates were incubated with streptavidin-Dynabeads-coupled-biotinylated miR-21 duplex for biotinylated RNA-streptavidin pull down assay(E).

Zhao_Fig.S3

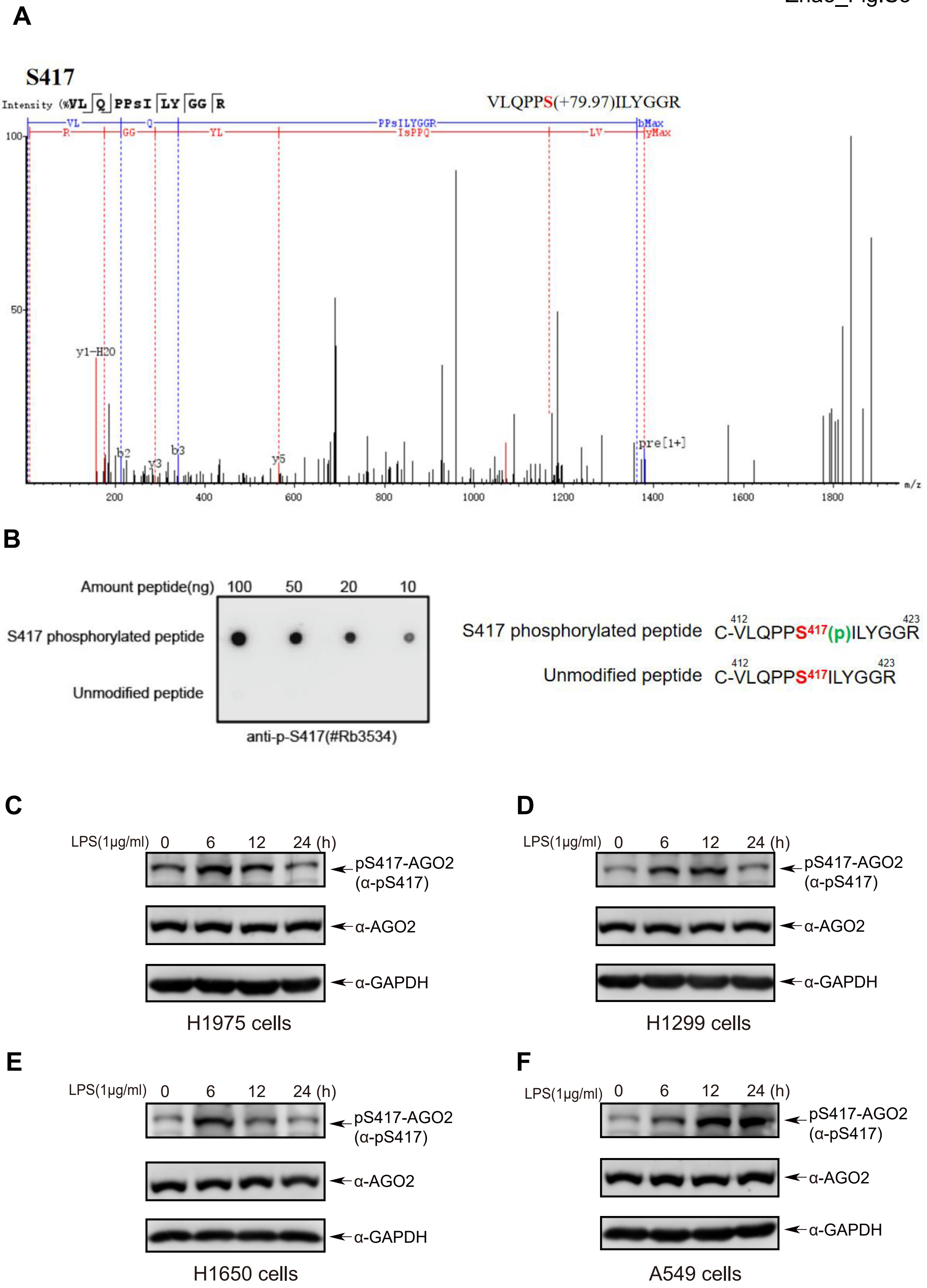
A
S417
Intensity (%)
VLQPPS(+79.97)ILYGGR
y1-H2O
b2
y3
b3
y5
pre[1+]
bMax
yMax
m/z
B
Amount peptide(ng)
100
50
20
10
S417 phosphorylated peptide
Unmodified peptide
anti-p-S417(#Rb3534)
S417 phosphorylated peptide C-VLQPPS417(p)ILYGGR
Unmodified peptide C-VLQPPS417ILYGGR
C
LPS(1μg/ml) 0 6 12 24 (h)
pS417-AGO2 (α-pS417)
α-AGO2
α-GAPDH
H1975 cells
D
H1299 cells
E
H1650 cells
F
A549 cells

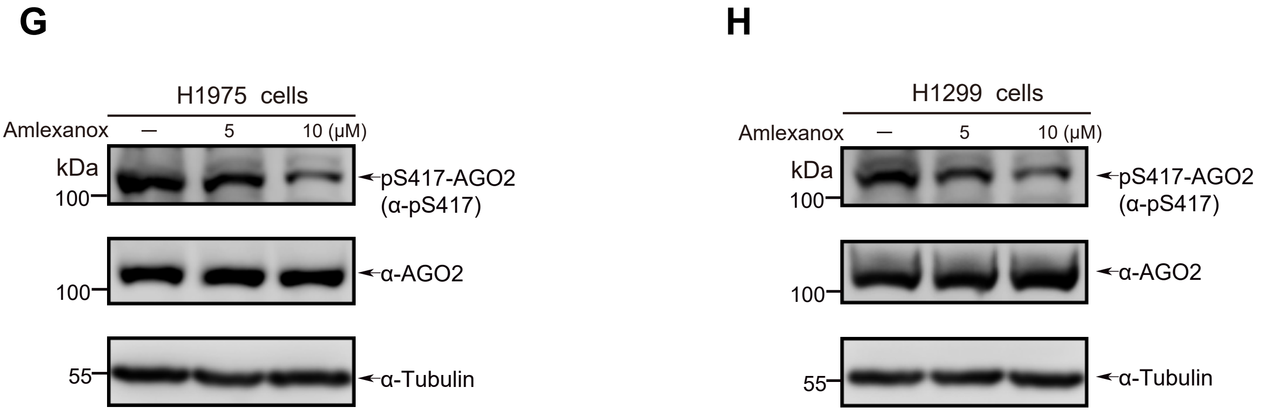

Figure.S3. S417 is phosphorylation site of AGO2 catalyzed by TBK1, Related to Figure 3.
(A) S417 is identified as the AGO2 phosphorylation site by mass spectrometry analysis. AGO2 protein from 293T cells co-transfected with Flag-AGO2 and TBK1 was purified by IP with anti-Flag antibody, then subjected to 8% SDS-PAGE gel, followed by staining with Coomassie brilliant blue. The band of Flag-AGO2 was cut and digested for the mass spectrometry analysis. (B) Characterization of specific pS417-AGO2 antibody. Specificity of pS417-AGO2 antibody was determined by dot blot assay (left panel). Different amounts of phospho-S417 or unmodified peptides were spotted on nitrocellulose membrane, and then detected with specific pS417-AGO2 antibody. (C-F) H1975(C), H1299 (D), H1650(E),and A549 (F) cells treated with LPS (1 μg/ml) for the indicated times were lysed for Western blotting analysis by using the specific pS417-AGO2 antibody. (G-H) H1975 (G) or H1299 (H) cells treated with Amlexanox for 12 h before harvested. Lysates were used for Western blotting analysis with specific pS417-AGO2 antibody.

Zhao_Fig.S4

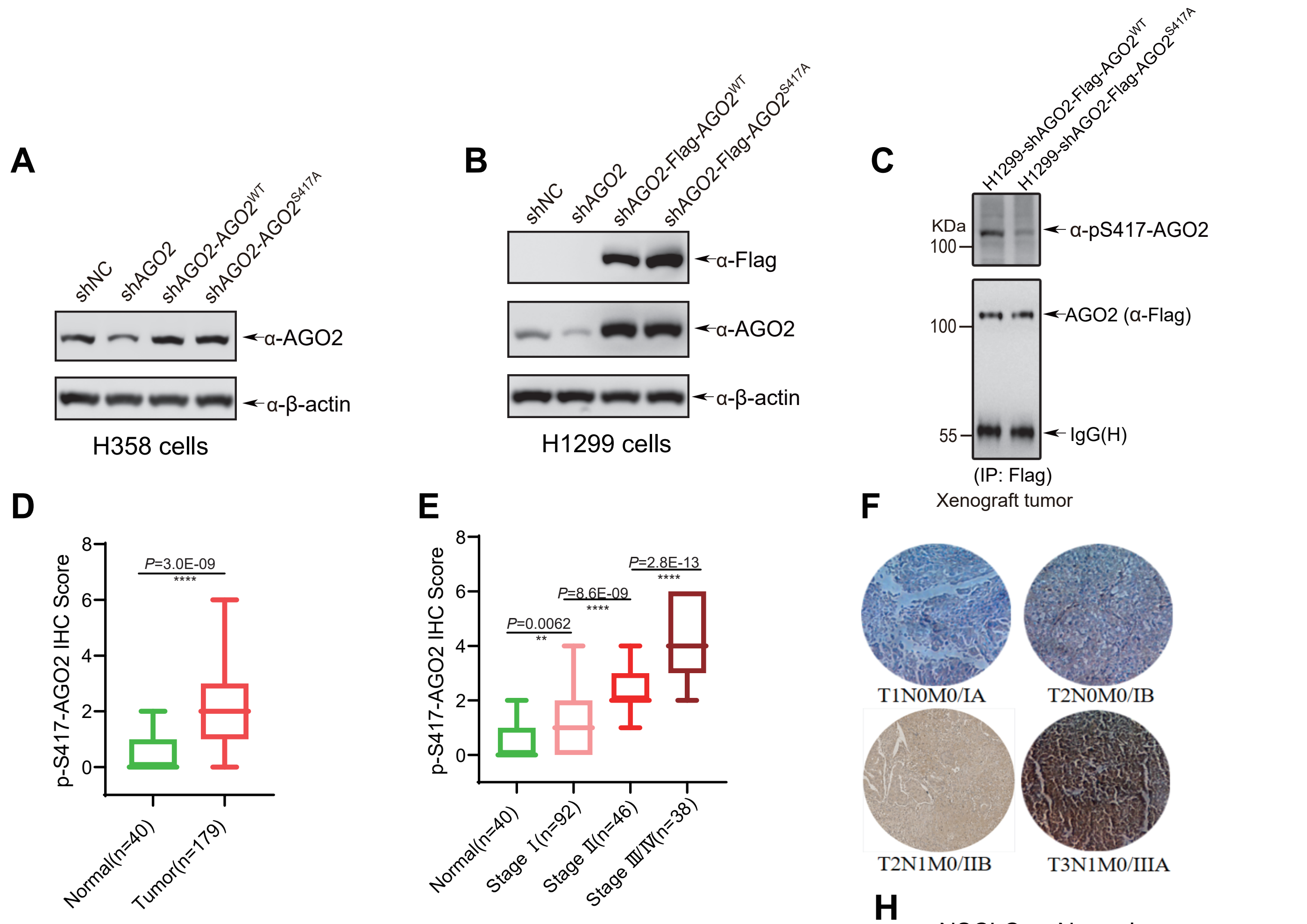

G

Factor	Patient Number	Fold change (NSCLC vs Normal)	P value	Significance
AGO2 protein	60	-1.08	P=0.598	ns
pS417-AGO2	60	1.62	P<0.0001	****

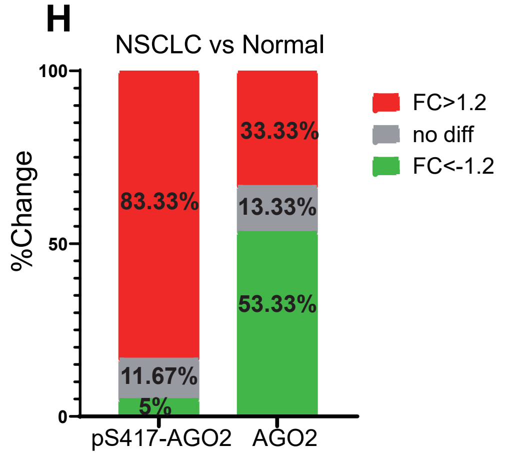

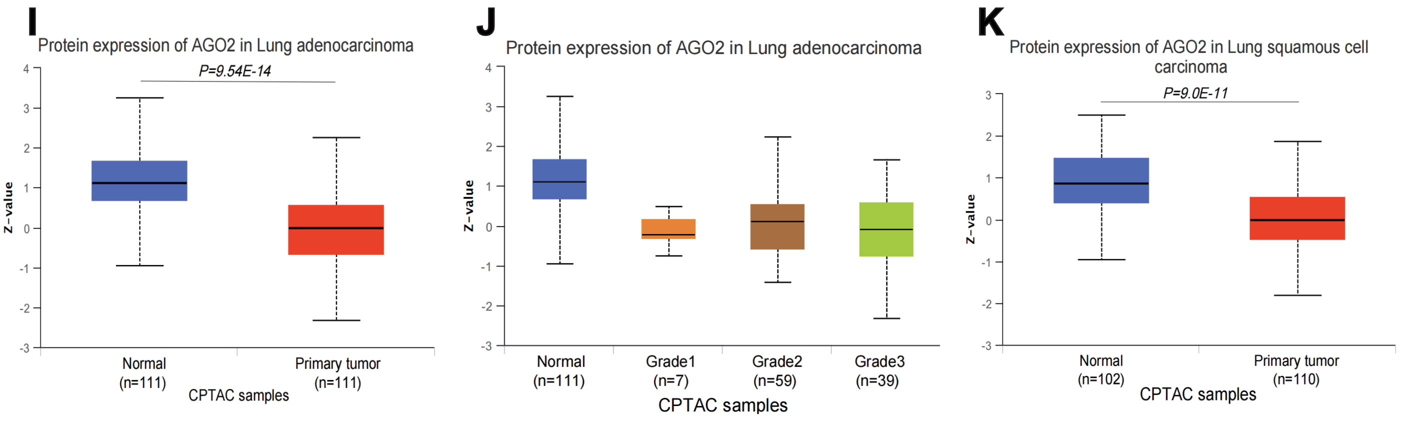

Figure S4. Phosphorylation of AGO2 at S417 promotes NSCLC progression, Related to Figure 4.
(A-B) Lysates from H358 (A) or H1299 (B) stable cell lines were immunoblotted with indicated antibodies. (C)The phosphorylation levels of AGO2 in xenografted tumors with H1299-shAGO2-Flag-AGO2WT and H1299-shAGO2-Flag-Flag-AGO2S417A were analyzed by Western blotting analysis with the specific pS417-AGO2 antibody. (D-F) IHC detection for pS417-AGO2 in lung cancer tissue arrays. (D) IHC staining scores for pS417-AGO2 levels in normal tissues (n=40) and lung cancer tissues (n=179) were shown.Statistical analysis was performed using unpaired t test, ****$P < 0.0001$. (E) IHC staining scores for pS417-AGO2 in normal tissues and pathological sub-stage lung cancers were shown. The lines of box plots in and represent the median, first and third quartiles, the whiskers denote the minima and maxima. Statistical analysis was performed using one-way ANOVA. **$P < 0.01$ *and* ****$P < 0.0001$. (F) Representative images of IHC staining for pS417-AGO2 levels in normal tissues and pathological sub-stages lung cancer tissues. (G-H) IHC staining of pS417-AGO2 and AGO2 protein in normal tissues (n=60) and NSCLC tissues (n=60).(G) The fold change of pS417-AGO2 and AGO2 protein between normal tissues and NSCLC tissues were shown. (H) The proportion of changes in pS417-AGO2 and AGO2 protein between normal tissues and NSCLC samples were calculated. (I-K) Analysis of AGO2 protein levels in the public database of Clinical Proteomic Tumor Analysis Consortium (CPTAC). (I) AGO2 protein levels in normal tissues (n=111) and lung adenocarcinoma (n=111) were shown. (J) AGO2 protein levels in pathological sub-stage of lung adenocarcinoma. (I) AGO2 protein levels in normal tissues (n=102) and lung squamous cell carcinoma samples (n=110) were shown. *Z*-values represent standard deviations from the median across samples for the given cancer type. Log2 Spectral count ratio values from CPTAC were first normalized within each sample profile, then normalized across samples.

Zhao_Fig. S5

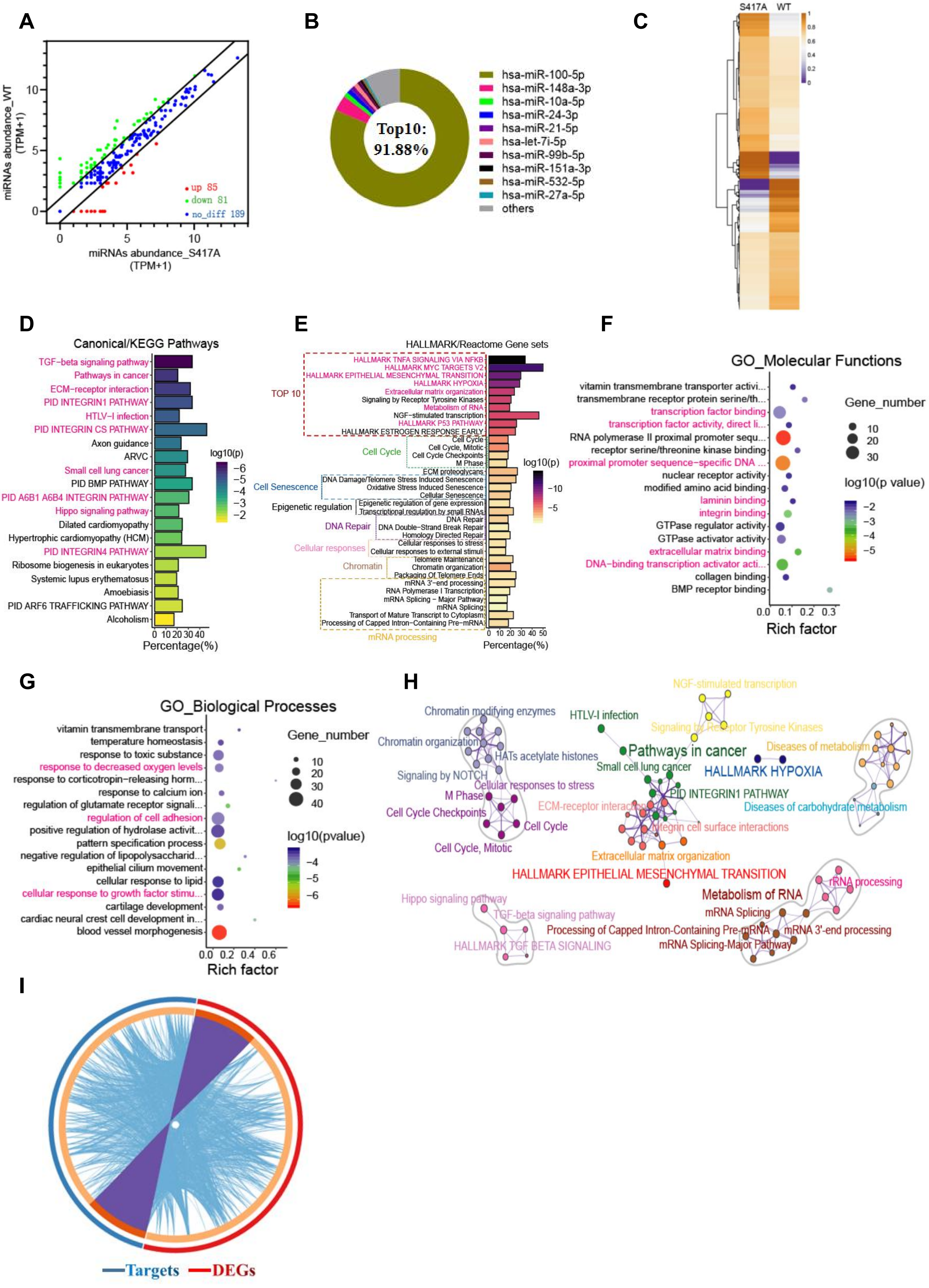
A
miRNAs abundance_WT (TPM+1)
miRNAs abundance_S417A (TPM+1)
up 85
down 81
no_diff 189
B
Top10: 91.88%
hsa-miR-100-5p
hsa-miR-148a-3p
hsa-miR-10a-5p
hsa-miR-24-3p
hsa-miR-21-5p
hsa-let-7i-5p
hsa-miR-99b-5p
hsa-miR-151a-3p
hsa-miR-532-5p
hsa-miR-27a-5p
others
C
S417A
WT
D
Canonical/KEGG Pathways
TGF-beta signaling pathway
Pathways in cancer
ECM-receptor interaction
PID INTEGRIN1 PATHWAY
HTLV-I infection
PID INTEGRIN CS PATHWAY
Axon guidance
ARVC
Small cell lung cancer
PID BMP PATHWAY
PID A6B1 A6B4 INTEGRIN PATHWAY
Hippo signaling pathway
Dilated cardiomyopathy
Hypertrophic cardiomyopathy (HCM)
PID INTEGRIN4 PATHWAY
Ribosome biogenesis in eukaryotes
Systemic lupus erythematosus
Amoebiasis
PID ARF6 TRAFFICKING PATHWAY
Alcoholism
log10(p)
Percentage(%)
E
HALLMARK/Reactome Gene sets
TOP 10
HALLMARK TNFA SIGNALING VIA NFKB
HALLMARK MYC TARGETS V2
HALLMARK EPITHELIAL MESENCHYMAL TRANSITION
HALLMARK HYPOXIA
Extracellular matrix organization
Signaling by Receptor Tyrosine Kinases
Metabolism of RNA
NGF-stimulated transcription
HALLMARK P53 PATHWAY
HALLMARK ESTROGEN RESPONSE EARLY
Cell Cycle
Cell Cycle
Cell Cycle, Mitotic
Cell Cycle Checkpoints
M Phase
ECM proteoglycans
Cell Senescence
DNA Damage/Telomere Stress Induced Senescence
Oxidative Stress Induced Senescence
Cellular Senescence
Epigenetic regulation
Epigenetic regulation of gene expression
Transcriptional regulation by small RNAs
DNA Repair
DNA Repair
DNA Double-Strand Break Repair
Homology Directed Repair
Cellular responses
Cellular responses to stress
Cellular responses to external stimuli
Chromatin
Telomere Maintenance
Chromatin organization
Packaging Of Telomere Ends
mRNA 3'-end processing
RNA Polymerase I Transcription
mRNA Splicing - Major Pathway
mRNA Splicing
Transport of Mature Transcript to Cytoplasm
Processing of Capped Intron-Containing Pre-mRNA
mRNA processing
log10(p)
Percentage(%)
F
GO_Molecular Functions
vitamin transmembrane transporter activi...
transmembrane receptor protein serine/th...
transcription factor binding
transcription factor activity, direct li...
RNA polymerase II proximal promoter sequ...
receptor serine/threonine kinase binding
proximal promoter sequence-specific DNA ...
nuclear receptor activity
modified amino acid binding
laminin binding
integrin binding
GTPase regulator activity
GTPase activator activity
extracellular matrix binding
DNA-binding transcription activator acti...
collagen binding
BMP receptor binding
Gene_number
log10(p value)
Rich factor
G
GO_Biological Processes
vitamin transmembrane transport
temperature homeostasis
response to toxic substance
response to decreased oxygen levels
response to corticotropin-releasing horm...
response to calcium ion
regulation of glutamate receptor signali...
regulation of cell adhesion
positive regulation of hydrolase activit...
pattern specification process
negative regulation of lipopolysaccharid...
epithelial cilium movement
cellular response to lipid
cellular response to growth factor stimu...
cartilage development
cardiac neural crest cell development in...
blood vessel morphogenesis
Gene_number
log10(pvalue)
Rich factor
H
NGF-stimulated transcription
Chromatin modifying enzymes
HTLV-I infection
Signaling by Receptor Tyrosine Kinases
Chromatin organization
Pathways in cancer
Diseases of metabolism
HATs acetylate histones
Small cell lung cancer
HALLMARK HYPOXIA
Signaling by NOTCH
Cellular responses to stress
M Phase
PID INTEGRIN1 PATHWAY
ECM-receptor interaction
Diseases of carbohydrate metabolism
Cell Cycle Checkpoints
Cell Cycle
Integrin cell surface interactions
Cell Cycle, Mitotic
Extracellular matrix organization
HALLMARK EPITHELIAL MESENCHYMAL TRANSITION
rRNA processing
Hippo signaling pathway
Metabolism of RNA
mRNA Splicing
TGF-beta signaling pathway
Processing of Capped Intron-Containing Pre-mRNA
mRNA 3'-end processing
HALLMARK TGF BETA SIGNALING
mRNA Splicing-Major Pathway
I
Targets
DEGs

Figure S5. The phosphorylation of AGO2 at S417 regulates gene expression by affecting miRNA loading, Related to Figure 5.

(A) Scatter plots showing the differentially expressed miRNA with fold change ⩾ 1.5 in stable cells H1299-shAGO2-Flag-AGO2^{WT} and H1299-shAGO2-Flag-AGO2^{S417A}. (B) Pie graph showing the abundance of miRNAs. (C) Heatmap showing the differentially expressed genes (DEGs) with log2 fold change≥0.5. The colors are according to z-score. (D) KEGG/Canonical Pathways enrichment analysis for the differentially expressed genes. (E) Hallmark/Reactome Gene sets analysis for the differentially expressed genes. (F-G) Gene ontology (GO) analysis of the differentially expressed genes. (H) Representation of the most enriched pathways within the differentially expressed genes. (I) The circle plot visualizing the overlap of targets of the top 10 most abundant miRNAs and DEGs. On the outer, each represents the identity of each gene list. Dark orange color and purple lines represent the same genes which appear in the two groups. Light orange color represents the unique genes to the list, and blue lines link the genes where they fall into the same enriched pathways.

Zhao_Fig. S6

A

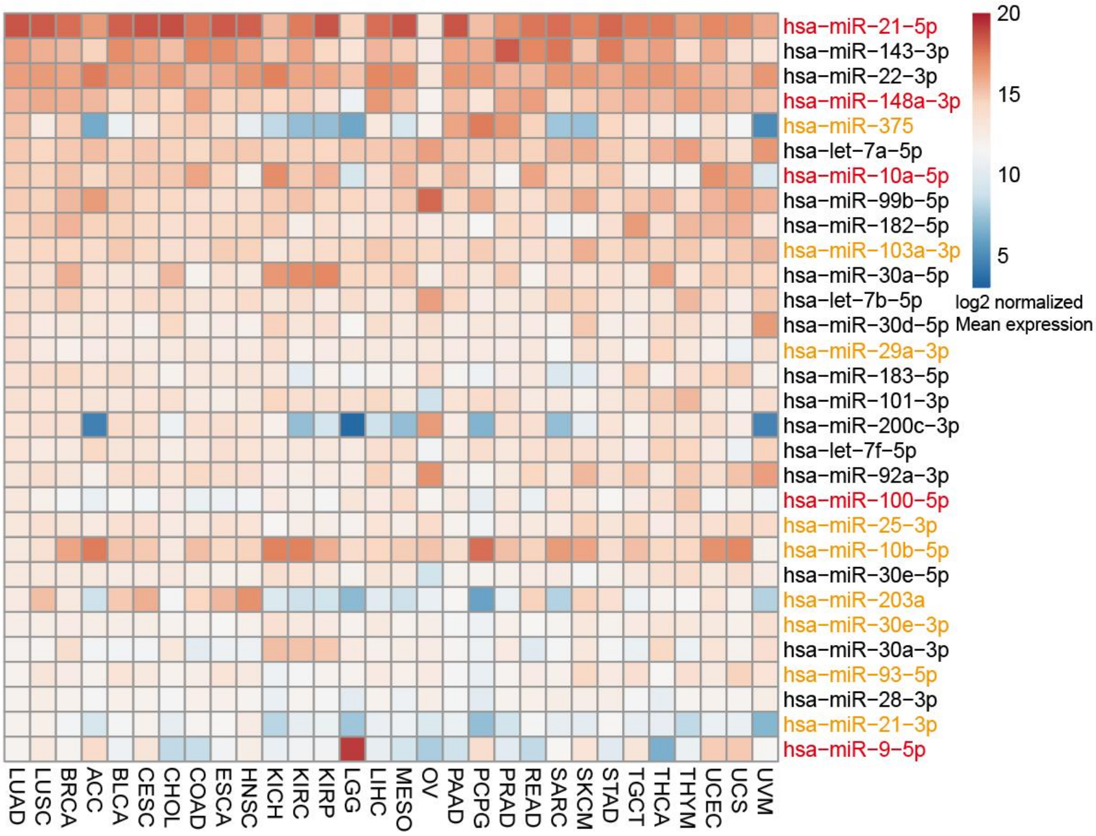

B

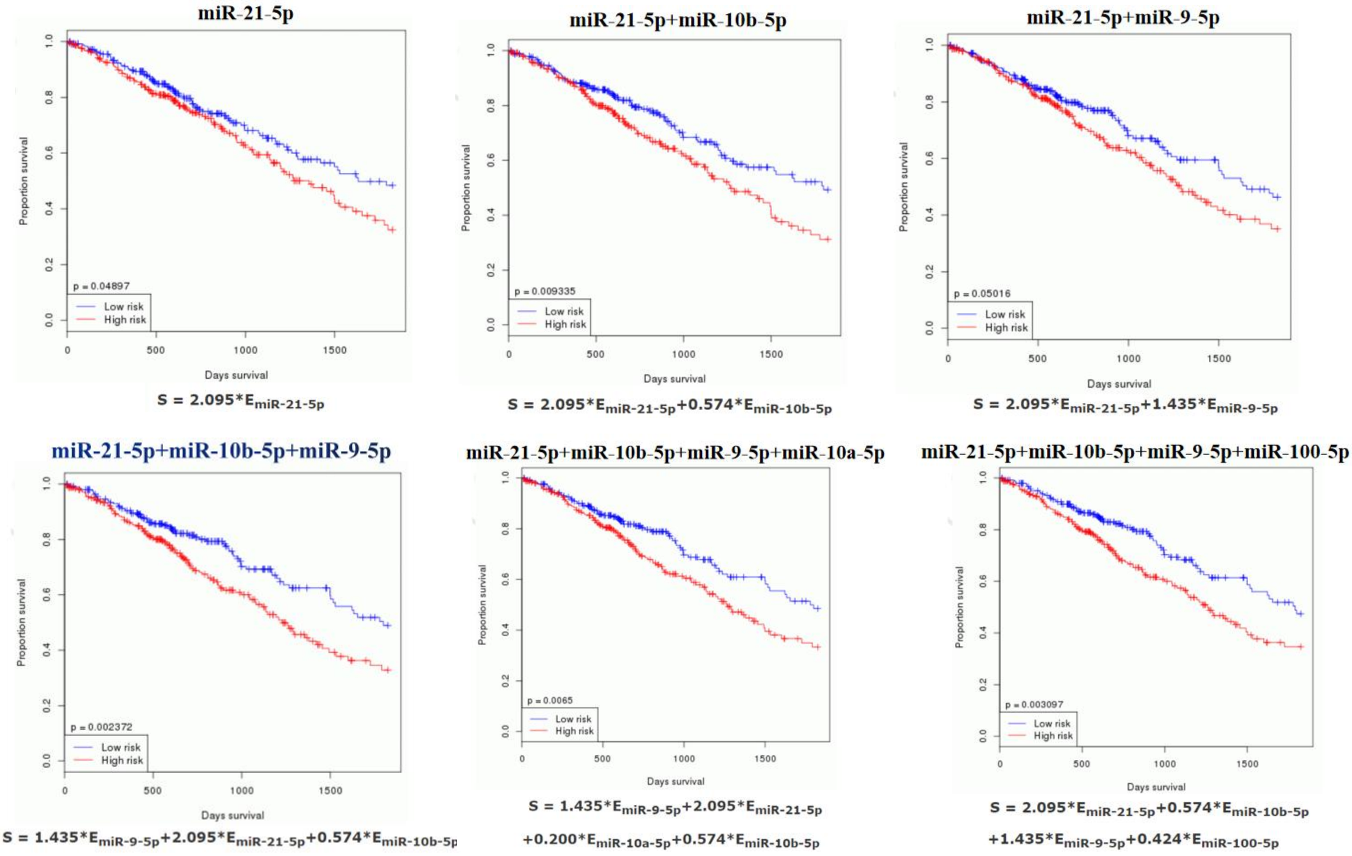

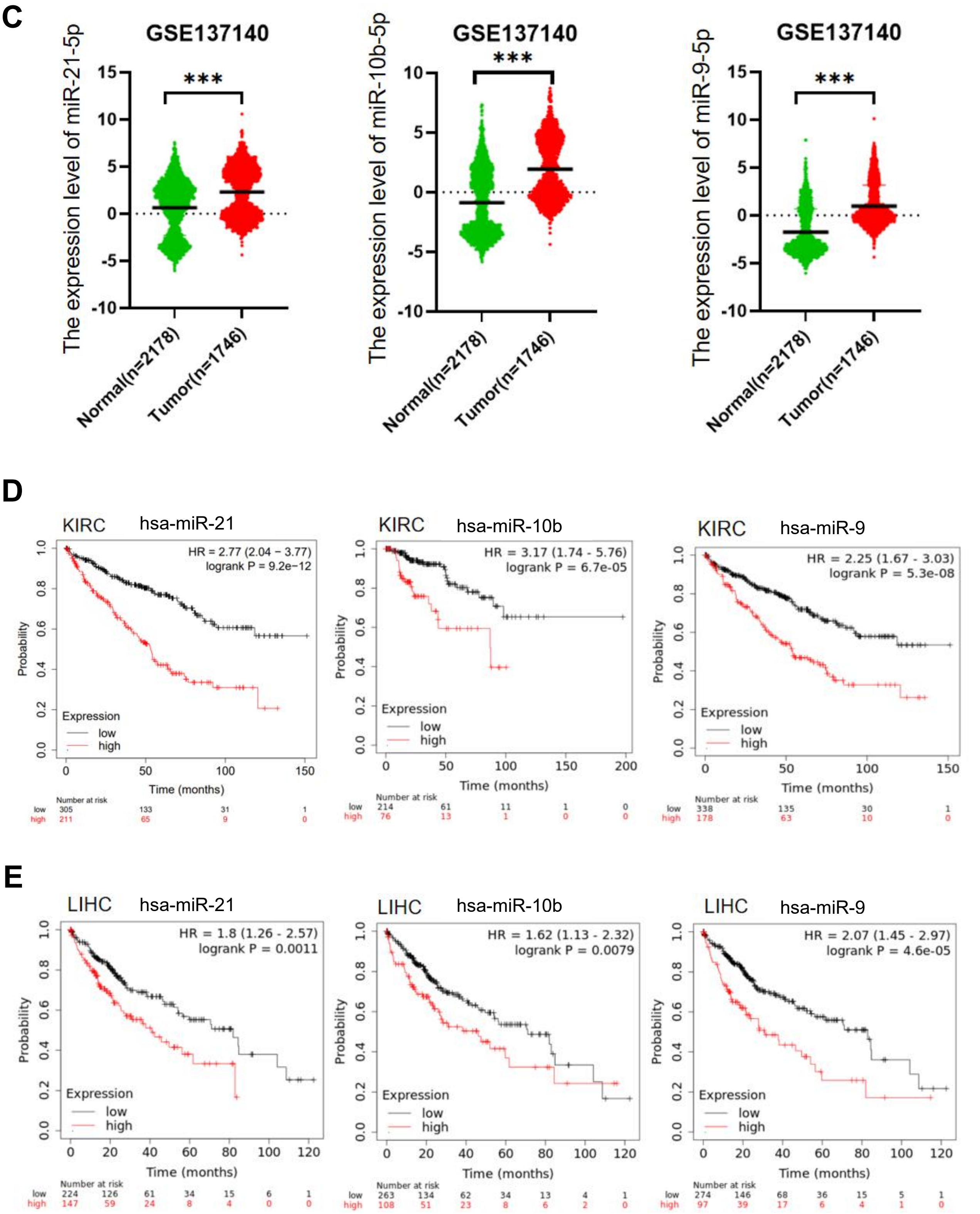

Figure S6. The high-abundance miRNAs were regulated by pS417-AGO2, Related to Figure 6. (A) Heatmap showing the most abundant top30 miRNAs in the 30 tumor types from TCGA database. (B) The optimal miRNA combination of miRNA-based therapeutic strategies for NSCLC patients was developed by univariate cox analysis. (C) The levels of miR-21, miR-10b and miR-9 in the clinical lung cancer datasets from the Gene Expression Omnibus (GEO) (accession GSE137140). (D-E) The correlation between the expression levels of miRNA and the survival of KIRC (D) and LIHC (E) patients was analyzed by the Kaplan-Meier analysis.

Zhao_Fig.S7

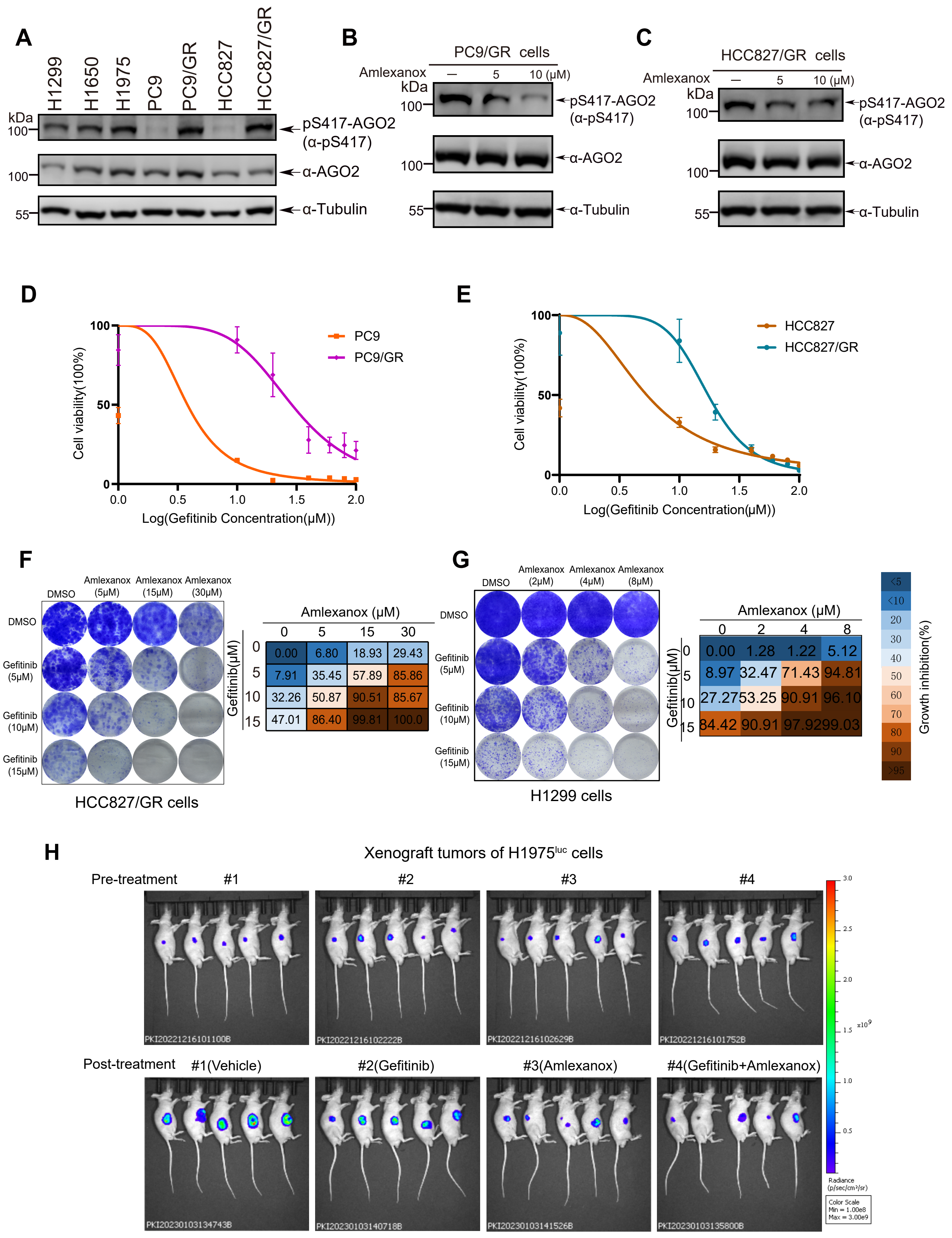
A
H1299
H1650
H1975
PC9
PC9/GR
HCC827
HCC827/GR
kDa
100
pS417-AGO2
(α-pS417)
α-AGO2
55
α-Tubulin
B
PC9/GR cells
Amlexanox — 5 10 (μM)
C
HCC827/GR cells
D
Cell viability(100%)
Log(Gefitinib Concentration(μM))
PC9
PC9/GR
E
HCC827
HCC827/GR
F
DMSO
Amlexanox (5μM)
Amlexanox (15μM)
Amlexanox (30μM)
Gefitinib (5μM)
Gefitinib (10μM)
Gefitinib (15μM)
HCC827/GR cells
Amlexanox (μM)
Gefitinib(μM)
0 5 15 30
0: 0.00 6.80 18.93 29.43
5: 7.91 35.45 57.89 85.86
10: 32.26 50.87 90.51 85.67
15: 47.01 86.40 99.81 100.0
G
Amlexanox (2μM)
Amlexanox (4μM)
Amlexanox (8μM)
H1299 cells
0 2 4 8
0: 0.00 1.28 1.22 5.12
5: 8.97 32.47 71.43 94.81
10: 27.27 53.25 90.91 96.10
15: 84.42 90.91 97.92 99.03
Growth inhibition(%)
H
Xenograft tumors of H1975luc cells
Pre-treatment
#1 #2 #3 #4
Post-treatment
#1(Vehicle)
#2(Gefitinib)
#3(Amlexanox)
#4(Gefitinib+Amlexanox)
Radiance (p/sec/cm²/sr)
Color Scale Min = 1.00e8 Max = 3.00e9

Zhao_Fig.S7

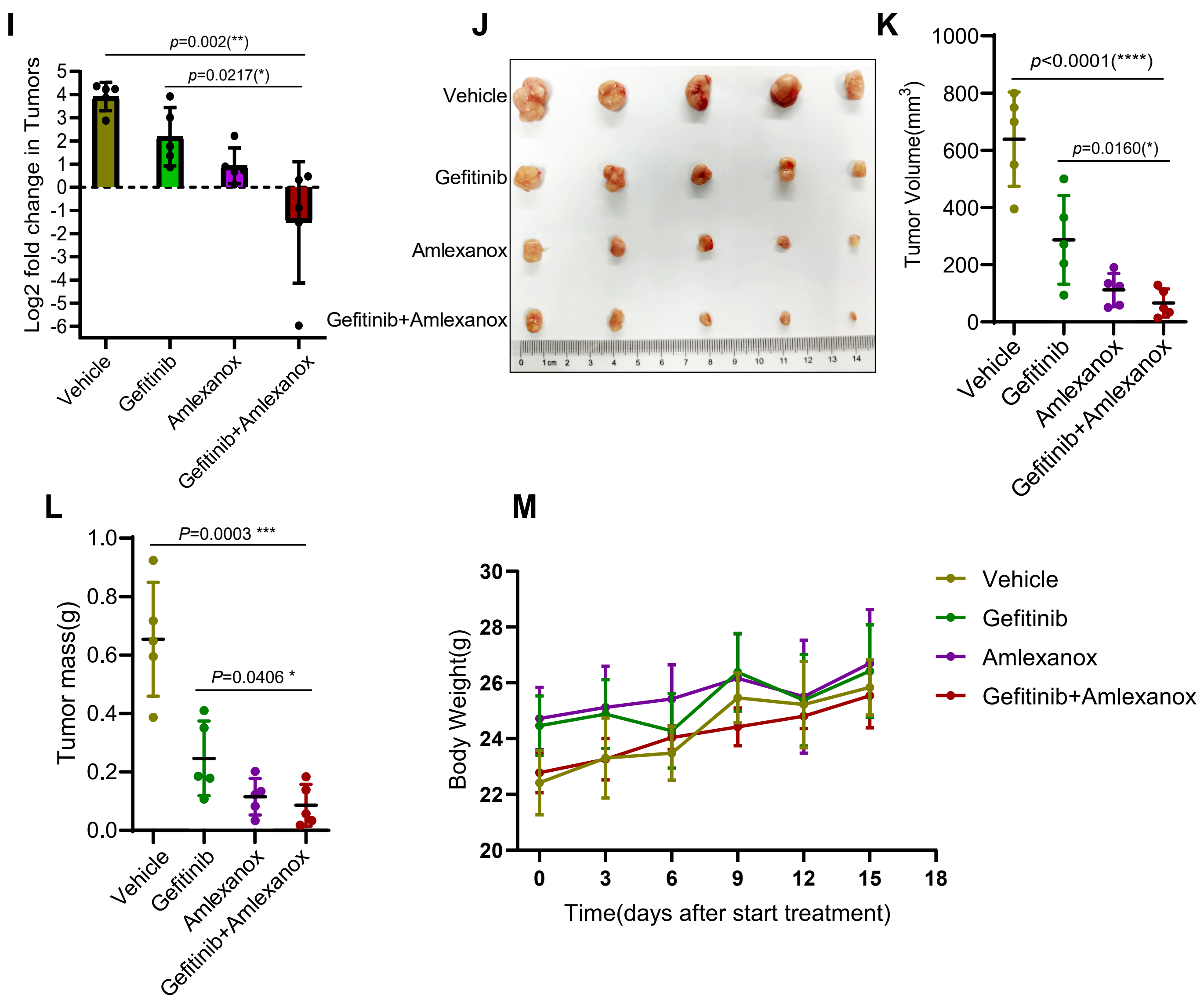

Figure.S7. The combination therapy of Amlexanox overcomes resistance to Gefitinib, Related to Figure 7. (A) The phosphorylation levels of AGO2 in the indicated NSCLC cell lines were analyzed by Western blotting analysis with specific anti-pS417-AGO2 and anti-AGO2 antibodies.(B-C) PC9/GR(B) or HCC827/GR (C) cells treated with Amlexanox for 12 h before harvested. Lysates were used for Western blotting analysis with the indicated antibodies. (D) The viabilities of PC9 and PC9/GR cells after 48 h of treatment with gefitinib were analyzed. (E) The viabilities of HCC827 and HCC827-GR after 48 h of treatment with gefitinib were analyzed. (F-G) HCC827/GR (F) and H1299 (G) cells were treated with Gefitinib, Amlexanox or their combination at the indicated concentrations. Cells were fixed and stained after 10-12 days. Representative data from three independent experiments. (H-L) Mice were subcutaneously injected with 1×10^6 $H1975^{luc}$ cells. Once tumors reached an average of 5 mm × 5 mm (14 days), the mice were treated with Gefitinb (20 mg/kg) by oral gavage, Amlexanox (25 mg/kg) by intraperitoneal injection, or combination therapy for every 3 days.(H) Representative bioluminescence images of mice before and after treatment. The luciferase signal intensity was monitored in vivo by bioluminescence imaging, and fold change in tumor pre or post-treatment was analysed (I). Xenograft tumors were dissected (J), tumor volume (K) and tumor weights (L) were assessed after treatment. Data were presented as mean ± SD, n = 5. *$^{*}P < 0.05$, $^{***}P < 0.001$ and $^{****}P < 0.0001$*. (M) The mouse body weight during treatment were monitored.
